# Protecting Great Barrier Reef resilience through effective management of crown-of-thorns starfish outbreaks

**DOI:** 10.1371/journal.pone.0298073

**Published:** 2024-04-24

**Authors:** Samuel A. Matthews, David H. Williamson, Roger Beeden, Michael J. Emslie, Rickard T. M. Abom, Daniel Beard, Mary Bonin, Peran Bray, Adriana R. Campili, Daniela M. Ceccarelli, Leanne Fernandes, Cameron S. Fletcher, Dan Godoy, Christopher R. Hemingson, Michelle J. Jonker, Bethan J. Lang, Sheriden Morris, Enrique Mosquera, Gareth L. Phillips, Tane H. Sinclair-Taylor, Sascha Taylor, Dieter Tracey, Jennifer C. Wilmes, Richard Quincey

**Affiliations:** 1 Great Barrier Reef Marine Park Authority, Townsville, QLD, Australia; 2 Australian Institute of Marine Science, Townsville, QLD, Australia; 3 Reef and Rainforest Research Centre, Cairns, QLD, Australia; 4 INLOC Group, Cairns, QLD, Australia; 5 Great Barrier Reef Foundation, Brisbane City, QLD, Australia; 6 CSIRO, Townsville, QLD, Australia; 7 Blue Planet Marine, Canberra, ACT, Australia; 8 The University of Texas at Austin, Marine Science Institute, Port Aransas, Texas, United States of America; 9 The University of New South Wales, Sydney, NSW, Australia; 10 ARC Centre of Excellence, James Cook University, Townsville, QLD, Australia; 11 Pacific Marine Group, Townsville, QLD, Australia; 12 Association of Marine Park Tourism Operators Ltd, Cairns, QLD, Australia; 13 Queensland Department of Environment and Science, Queensland Parks and Wildlife Service and Partnerships (Marine Parks), Brisbane, Queensland, Australia; Secretariat of the Pacific Community, NEW CALEDONIA

## Abstract

Resilience-based management is essential to protect ecosystems in the Anthropocene. Unlike large-scale climate threats to Great Barrier Reef (GBR) corals, outbreaks of coral-eating crown-of-thorns starfish (COTS; *Acanthaster* cf. *solaris*) can be directly managed through targeted culling. Here, we evaluate the outcomes of a decade of strategic COTS management in suppressing outbreaks and protecting corals during the 4^th^ COTS outbreak wave at reef and regional scales (sectors). We compare COTS density and coral cover dynamics during the 3^rd^ and 4^th^ outbreak waves. During the 4th outbreak wave, sectors that received limited to no culling had sustained COTS outbreaks causing significant coral losses. In contrast, in sectors that received timely and sufficient cull effort, coral cover increased substantially, and outbreaks were suppressed with COTS densities up to six-fold lower than in the 3^rd^ outbreak wave. In the Townsville sector for example, despite exposure to comparable disturbance regimes during the 4^th^ outbreak wave, effective outbreak suppression coincided with relative increases in sector-wide coral cover (44%), versus significant coral cover declines (37%) during the 3^rd^ outbreak wave. Importantly, these estimated increases span entire sectors, not just reefs with active COTS control. Outbreaking reefs with higher levels of culling had net increases in coral cover, while the rate of coral loss was more than halved on reefs with lower levels of cull effort. Our results also indicate that outbreak wave progression to adjoining sectors has been delayed, probably via suppression of COTS larval supply. Our findings provide compelling evidence that proactive, targeted, and sustained COTS management can effectively suppress COTS outbreaks and deliver coral growth and recovery benefits at reef and sector-wide scales. The clear coral protection outcomes demonstrate the value of targeted manual culling as both a scalable intervention to mitigate COTS outbreaks, and a potent resilience-based management tool to “buy time” for coral reefs, protecting reef ecosystem functions and biodiversity as the climate changes.

## 1. Introduction

Anthropogenically-driven disturbances are superimposing additional stressors upon the natural cycles of decline and recovery inherent to coral reef ecosystems [1] major drivers of coral mortality on Indo-Pacific coral reefs are cyclones, coral bleaching events, and outbreaks of crown-of-thorns starfish (COTS) *Acanthaster* cf. *solaris* [2–6]. Climate driven disturbances are increasing in frequency, intensity, and spatial scale, subjecting many coral reef ecosystems to escalating cumulative impacts with shorter disturbance-free recovery periods [2,3]. Ongoing chronic impacts (e.g. overfishing, pollution and sedimentation) compound acute disturbances, further limiting the capacity of reefs to recover and regain pre-disturbance levels of diversity, ecological function and productivity [4,5].

Strategic management interventions, such as pest and pollution management are becoming increasingly necessary to mitigate damage and assist the recovery of many ecosystems, including the Great Barrier Reef (GBR) [6,7]. The rapid increase in frequency and severity of recurrent disturbances has made resilience-based management (RBM) key to steering coral reefs through the Anthropocene [6,8,9]. The RBM approach identifies current and future drivers of environmental change to prioritise, implement, and adapt management actions that can assist ecosystems to resist and recover from cumulative stressors as the climate changes [9,10]. Of the three major drivers of coral mortality on the GBR, only COTS outbreaks can be directly mitigated via local management action [11–14].

The first COTS outbreak wave identified within the Great Barrier Reef Marine Park (Marine Park) was recorded on reefs offshore from Cairns in 1962 [15]. This was followed by three subsequent outbreak waves over the following decades, each persisting for 10–15 years and resulting in significant coral losses across much of the GBR [14–18]. Evidence shows that COTS outbreak waves originate on reefs in the northern region of the Marine Park (referred to as “the initiation box”) [14,19]. Larval dispersal from these primary outbreak reefs drives recruitment of successive cohorts, perpetuating waves of secondary outbreaks that progress both southward and northward (driven by the bifurcation of the North Vanuatu Jet [20]), through the Marine Park [14,21]. COTS outbreaks accounted for approximately 40% of the coral loss recorded on the GBR between 1985 and 2012, corresponding to a decline in coral cover of approximately -1.42% per year [13]. The same analysis indicated that even in the presence of coral bleaching and cyclone damage, prevention of COTS predation would have yielded a net increase in GBR-wide coral cover over the same period [13].

Since its establishment in 1975, the Great Barrier Reef Marine Park Authority (Reef Authority), in partnership with the tourism industry and research community, has been evaluating the severity of COTS outbreaks and effective options for their management [22,23]. The first COTS management interventions mostly involved manual removal of starfish, with limited impact beyond site-scales or on long-term outbreak dynamics [24]. The first COTS Control Program (the Program) began in 2002 in response to the 3^rd^ outbreak wave, deploying culling via injection of COTS with multiple shots of sodium bisulphate solution, that proved efficient and effective albeit at site scales [25]. Although significant numbers of COTS were culled and coral was protected across many high-value tourism sites, the Program did not have sufficient resourcing to be deployed at reef and regional scales. Building upon these foundations, the Program was remobilised with increased resources in 2012 in response to the emergence of the 4^th^ outbreak wave. Importantly, dedicated COTS research programs and iterative testing within the Program led to the development of ‘single shot’ injection culling techniques, using either ox bile salt or household vinegar solutions, to deliver a step-change in culling efficiency that made large scale COTS control operations feasible [26,27]. Furthermore, systematic reef monitoring programs, combined with the development of COTS and coral larval dispersal and connectivity models, substantially improved our understanding of COTS outbreak dynamics enabling precise selection of reefs for COTS control [28,29].

Reef managers, researchers, Program contractors, and the tourism industry and operators have worked in partnership over the past decade to deliver COTS control operations at increasing spatial scales. The spectre of climate change and the reality of mounting cumulative impacts has driven the Program’s development, with increased resourcing and on-water capacity from 1–2 dedicated vessels from 2012–2018, to 5–7 vessels since 2018. Importantly this expansion of resources (~300%) has enabled proactive surveillance and targeted culling across hundreds of reefs and coincided with the full implementation of a tailored Integrated Pest Management (IPM) framework in 2018 [30,31]. This IPM framework was the culmination of a decade of COTS research, primarily funded under the National Environmental Science Program (NESP), which improved the efficacy and efficiency of COTS control operations by ~63% [32]. These advances and outcomes established GBR-wide COTS control as a core priority of both the Reef 2050 Plan [33] and the Blueprint for Resilience [34], and underpin the $161.4m Australian government investment in the Program from 2022 to 2030. Long term funding has improved Program continuity and enhanced the expertise of contractors and vessel crew to control COTS more efficiently and safely. Increased capacity, coupled with technological advances, experienced crew and improved application methods, has significantly enhanced the opportunity to go beyond the protection of individual high-value (predominantly tourism) sites. The Program has evolved to target the protection of a network of ecologically and economically significant reefs to deliver regional (sector) scale outbreak suppression and coral protection outcomes. Indeed, targeted COTS control on the GBR represents an ecosystem scale application of resilience-based management [9] consistent with the recommendations for managing coral reefs in the Anthropocene [6].

The Program has been implemented consistent with the objectives of the COTS Strategic Management Framework, that describes a theoretical hierarchy of objectives designed to be applied sequentially to maximise coral protection across the Marine Park [23] (S1 Fig). Prevention is the first order objective, with the management action being to mitigate the environmental factors that contribute to the initiation of a primary outbreak wave (e.g. managing overfishing of predators, improving water quality). If surveillance indicates that a primary outbreak is underway, then suppression (i.e. containment) of the outbreak wave is the secondary objective, followed by the protection of coral at individual reefs if suppression cannot be achieved at regional scales. The ability to achieve higher order objectives of regional suppression likely depends upon the timeliness of action and the ability to allocate and sustain sufficient culling resources. Given that the Program was remobilised two years after the initiation of the primary outbreak in 2010, prevention was not possible. Management actions therefore initially focused on suppression of the primary outbreak in the early years of the Program (e.g. 2012–2015) and thereafter focused on the protection of individual high value reefs. Protecting coral from COTS predation has been the fundamental goal of the Program throughout the 4th outbreak wave.

The Program draws upon multiple empirical and modelled data, and extensive field intelligence, to identify ‘priority reefs’ that have high ecological and/or economic value and are vulnerable to COTS at various stages of an outbreak wave. Each year, a subset of priority reefs that are presently at risk from COTS, are logistically feasible to control and have adequate coral cover to sustain an outbreak, are identified as target reefs. Following a consultation process with Program partners and contractors, the target reefs become the operational focus for surveillance and culling in that year. The IPM framework is then used to direct reef surveillance, detect COTS, and ensure sustained control effort is directed to the appropriate target reefs until COTS densities are reduced to ecologically sustainable levels (i.e., below densities where coral growth and recovery will outpace COTS predation) [35,36]. While there is evidence that culling effectively suppresses COTS outbreaks and protects coral at sites actioned on individual target reefs [37], there has been limited evaluation of evidence for effective suppression of outbreaks and protection of coral at the scale of individual reefs and no evaluation across reef regions (hereafter: sectors) [7]. We hypothesise that the magnitude of outbreak suppression and coral protection outcomes that can be achieved from COTS management depends upon (1) how early into an outbreak culling begins, (2) how much culling effort can be allocated and sustained over time, and (3) the cumulative impacts of other disturbances.

Herein, we evaluate the outbreak suppression and coral protection outcomes of the Program at reef and sector scales. Specifically, we: (1) evaluate the effectiveness of COTS control in reducing COTS densities and protecting coral cover relative to the timing of management action and the level of effort applied, and (2) determine if these management actions generated coral protection benefits at reef and regional (sectoral) scales. To evaluate these outcomes, we make use of a long-term monitoring dataset [38] Program data to compare the densities of COTS and change in coral cover between the 3^rd^ and 4^th^ outbreak waves at reefs within sectors. We also assess relative changes in coral cover over the course of the 4^th^ outbreak wave when the Program was fully operational and compare coral cover outcomes at individual reefs relative to the levels of culling effort that were applied. Our findings quantify the outcomes to date and identify opportunities for operational and strategic improvement of the Program.

## 2. Material and methods

### 2.1. The COTS control program and study location

**Fig 1:**
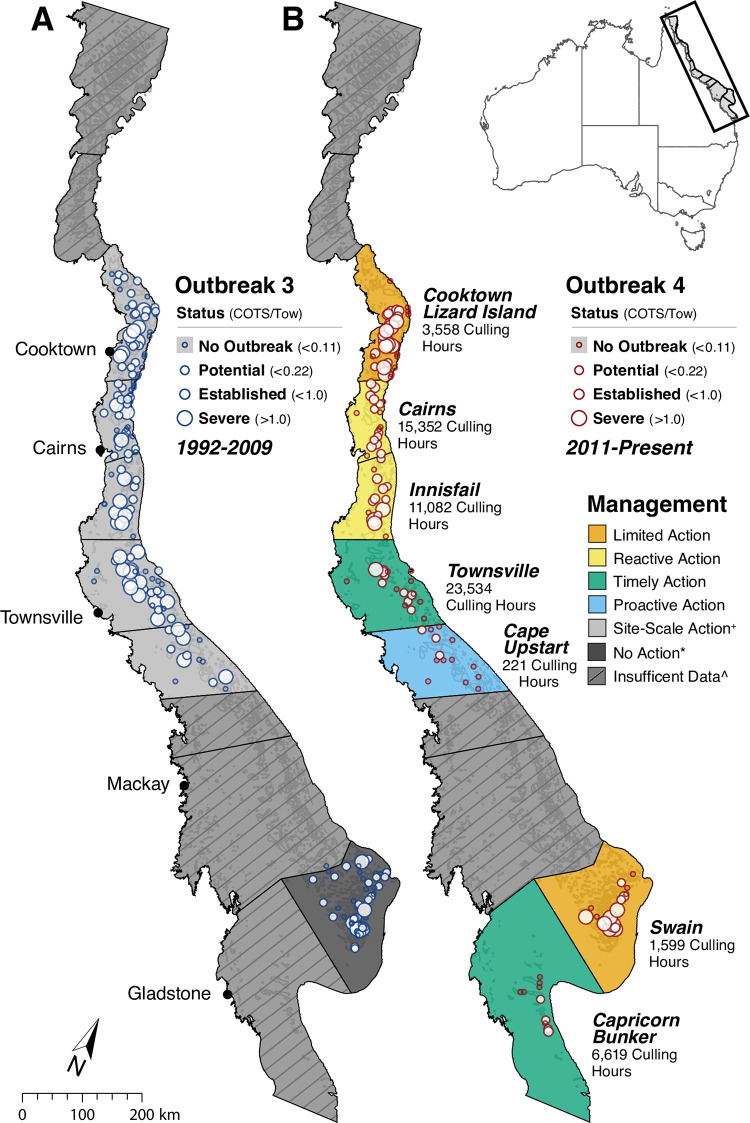
COTS outbreaks, spatial extent and management action. Maximum COTS density and the corresponding outbreak category at each reef during the 3^rd^ outbreak wave (A) (1992–2009) and the 4^th^ outbreak wave (B) (2011-Present) at LTMP reefs. Only reefs from sectors and outbreaks included in the following analysis are shown. Sector-scale colour coding depicts the management action taken during the 4^th^ outbreak wave with the number of culling hours (total of all cull divers bottom time) listed below the sector labels. ^+^ Site-Scale Action refers to COTS culling conducted at high-value tourism sites during the 3^rd^ outbreak wave, which had no discernible impact on sectoral level outbreak dynamics. * No Action refers to sectors where no control was implemented. ^ Insufficient Data reflects sectors where time series data was insufficient to determine Outbreak periods or no distinct outbreak wave was observed. These sectors have thus been excluded from these analyses. Sector outlines republished from AIMS: https://apps.aims.gov.au/reef-monitoring/sector/list under a CC BY license, with permission from Mike Emslie, AIMS under a CC BY license, original copyright 2023.

The Program is funded by the Australian Government and delivered via a partnership between the Great Barrier Reef Marine Park Authority (the Reef Authority; the governing body that manages the Marine Park), the Great Barrier Reef Foundation (GBRF), and the Reef and Rainforest Research Centre (RRRC). Field operations (surveillance and culling) is conducted by commercial contractors who supply their own vessels, equipment, divers and crew (for details of how the culling occurs *in situ*, please see the Supplemental Text). To date, the majority of culling effort has been focused on reefs spanning an approximately 600km stretch between Lizard Island and Cape Upstart (Fig 1B). This reflects the number of high value (economic and ecological) reefs in this area, their relative ease of access from major ports, and the intensity of secondary COTS outbreaks in this region [39]. More recently (since 2018), culling effort has also been directed toward the Southern region of the Marine Park on Capricorn-Bunker and Swain reefs. Here, we assess the outbreak suppression and coral protection outcomes of the Program across reefs distributed within sectors of the GBR spanning the majority of the Marine Park (Fig 1).

### 2.2. Estimates of coral cover and COTS abundance

Focal reefs within eleven sectors have been systematically monitored by the Australian Institute of Marine Science’s Long-Term Monitoring Program (LTMP) since 1986. The LTMP provides a spatially and temporally extensive dataset, collected following standard procedures for broad scale reef-wide estimates of coral cover and COTs abundance [40], that are used to assess status and trends in reef condition and to quantify the impacts of disturbance events and cumulative pressures on the Reef (S5 Fig). The LTMP dataset has enabled a robust evaluation of the outcomes of the Program on outbreak suppression and coral cover dynamics at the scale of individual reefs and across seven sectors in which control effort was deployed (Fig 1 and Table 1). The spatial hierarchy of sectors, reefs, and culling sites is presented in S2 Fig.

**Table 1:** COTS Management on the GBR.

Sector	Latitudinal span (°S)	3^rd^ Outbreak	4^th^ Outbreak	4^th^ Outbreak Culling Effort (Hours)	4^th^ Outbreak Management Action
*Cooktown/Lizard Island*	-14.2; -15.9	1994–2001	2011–2018	3,558 (5.7%)	Limited Action
*Cairns*	-15.9; -17.1	1998–2002	2013–2017	15,352 (24.8%)	Reactive action
*Innisfail*	-17.1; -18.3	1997–2004	2015–2021	11,082 (17.9%)	Reactive action
*Townsville*	-18.3; -19.4	1998–2008	2016-present	23,534 (37.9%)	Timely action
*Cape Upstart*	-19.4; -20.1	2002–2009	2020-present*	221 (0.4%)	Proactive action
*Swain*	-21.8; -23.0	1992–2006	2016-present	1,599 (2.6%)	Limited Action
*Capricorn Bunker*	-22.6; -24.9	Not recorded	2018-present	6,619 (10.7%)	Timely action

Sectors on the GBR managed under the COTS program, the outbreak duration within each sector, the management effort and action implemented within each sector. Management effort is only included on reefs also surveyed by the LMTP included in the analysis. * Indicates an expected outbreak based on historic outbreak patterns but has not yet eventuated.

Reefs are monitored using manta tow surveys, whereby a trained, experienced observer is towed behind a small vessel (5-6m) around the entire reef perimeter in a series of two-minute tows. Each tow is approximately 200m in length with a swathe width of ~10m (~2000 m^2^ survey area). At the end of each tow, observers record: 1) the estimated categorical coral cover, and 2) the number of COTS observed (note that additional variables are also recorded) [40]. Coral cover estimates and COTS abundances from each individual tow are then modelled to produce average reef level and sector-wide estimates for each survey year (see Section 2.5). The 36 years of monitoring has provided an extensive spatio-temporal dataset from which to examine coral cover and COTS dynamics from a total of 492 reefs spanning the Marine Park. These data formed the basis for assessing the subset of sector-wide trends in coral cover and COTS abundance from the seven sectors where control effort was deployed during the 4^th^ outbreak (n = 207 reefs).

COTS abundance data are also collected by trained observers via manta tow by the Reef Joint Field Management Program (RJFMP, 2012–2022) and by COTS Program contractors (2015–2022) using the same standard operating procedures as the LTMP. The data collected from these programs was used to supplement COTS density estimates from LTMP data where there were data gaps for specific reefs in specific years across the temporal series. Manta tow surveys conducted by the RJFMP and the Program are conducted prior to the initiation of cull operations. These RJFMP and Program pre-cull surveys provided a more representative estimate of COTS densities at specific reefs in certain years, particularly when LTMP surveys were conducted after the initiation of culling. If reefs were surveyed by multiple programs in a given year, the maximum COTS density recorded across all surveys was chosen as the representative sample for the reef.

### 2.3. Definition of sector-wide management action

Action taken by the Program between 2012 and 2022 was categorised based on the available resources that could be allocated to a sector as well as the timing of culling intervention relative to the onset of the outbreak (i.e. how far into the outbreak cycle did culling begin). To evaluate the overall efficacy of COTS control, sector level management actions were categorised by the culling activity conducted during the 4^th^ outbreak wave (Fig 1).

1. ***Proactive action*:** Pre-emptive targeted culling begins before COTS reach sector-wide outbreak densities (0.11 COTS/Tow), with the goal of delaying or preventing the onset of a sector-wide outbreak.
2. ***Timely action*:** Culling begins after sector-wide Potential Outbreak thresholds are reached (>0.11 COTS/Tow) but before more severe levels are reached. Program capacity is sufficient to suppress outbreaks at sector-wide scales.
3. ***Reactive action*:** Culling begins before Severe Outbreak (1 COTS/Tow) thresholds are reached, but Program capacity is insufficient to achieve outbreak suppression at sector-wide scales.
4. ***Limited Action*:** Culling begins before Severe Outbreak (1 COTS/Tow) thresholds are reached. Program capacity is insufficient to suppress outbreaks at sector-wide scales and/or control activities were initiated too late to prevent substantial coral loss. COTS outbreaks may become too severe to control at both the reef and sector-wide scales.

We then assess how effective different management actions were in reducing COTS densities and losses of coral cover during the 4^th^ outbreak wave compared to the 3^rd^ outbreak wave, when culling activity (when present) was restricted to protecting tourism sites. While successful at site-scales this culling did not result in discernible impacts to COTS outbreaks at reef or sector scales and are thus not included in the sector level analyses (Fig 1). Further detail is provided in the supplemental text.

### 2.4. Defining the outbreak period for each sector

To assess the performance of management action in controlling COTS and protecting coral, we needed to establish criteria that define the onset of an outbreak and the point at which COTS densities across the sector return below outbreak levels as defined by the Reef Authority and AIMS [23,41] (S3 Fig), whether due to active management or due to coral prey depletion. These criteria were:

- ***Outbreak inception*:** The sector-wide abundance of COTS must first exceed an initial threshold of >0.11 COTS/Tow (indicating a Potential Outbreak) and persist above this threshold for multiple years (≥2). This highlights a growing COTS population that warrants ongoing monitoring. For the Townsville and Swain sectors, the Established Outbreak threshold is used instead (>0.22 COTS/Tow) to reflect their 3-5-fold increased magnitude of COTS outbreaks [41] when compared to all other sectors.
- ***Outbreak escalation*:** When above 0.11 COTS/Tow, the density in following years then needs to exceed the Established Outbreak threshold of >0.22 COTS/Tow; at which, densities of COTS will reduce coral cover in the following years [11,41].
- ***Sector-wide outbreak*:** If -the two previous criteria are both met, a sector is classified as being impacted by a sector-wide “outbreak”.
- ***Outbreak decline*:** The outbreak is defined as ended when COTS density decreases below the potential threshold (0.11) for two consecutive years. If outbreak criteria are met, we additionally include one year prior to crossing the potential outbreak threshold (0.11) to capture the beginning phase of rapid population growth.

Using these criteria, we established the outbreak period for each sector (listed in Table 1). With these periods defined, we could assess how COTS density and coral cover changed over the course of an outbreak. The 4^th^ outbreak period defined for the Cape Upstart sector is based on ‘Proactive Action’. The rationale for this decision is provided in the supporting text (S1 Appendix).

### 2.5. Statistical modelling

Our aim was to evaluate the extent to which sector-specific management actions were able to reduce COTS densities and maintain or reduce the loss of coral cover. The effectiveness of each management action was assessed by comparing the densities of COTS and the change in coral cover between the 3^rd^ and 4^th^ outbreak waves within each sector, as well as the relative changes to coral cover over the course of the 4^th^ outbreak wave when the COTS Program was operational.

Modelling was conducted in R 4.2.2 [42]. All data handling was done using the ‘tidyverse’ packages [43]. All Bayesian models were fit using ‘brms’ [44] unless stated otherwise and GAMM models were fit using ‘mgcv’ package [44]. Goodness of fit and model residuals were explored using the ‘DHARMa’ package [45]. Estimated marginal means and pairwise comparisons were obtained using ‘emmeans’ [46] and the data was visualized using ‘ggplot2’ [47].

To track broad changes in coral cover, reef-wide manta tow observations were aggregated to form sector-wide estimates. A Bayesian hierarchical model was constructed that incorporated the varying survey effort amongst reefs and years so there was no temporal or spatial bias of sector wide coral cover estimates towards reefs that have been surveyed more frequently. Categorical estimates of hard coral cover for each individual two-minute tow were converted to category mid-points and used as the response variable. We then modelled temporal trends in hard coral cover in each individual sector employing generalised linear mixed models (glmm) using the *INLA* package [48]. Models were run separately for each sector with Year (categorical) as a fixed effect and Reef and Tow as random effects, using a beta error distribution. Goodness-of-fit diagnostics were confirmed and patterns in residuals explored using the ‘DHARMa’ package [45], indicating that the outputs of this model are robust, sector-wide, annual estimates of coral cover. These estimates were used to visualise general patterns of coral cover change through time within each sector.

From the original data set of 207 reefs that were used to generate the sector wide hard coral trajectories only reefs that had recorded COTS outbreaks above the Potential Outbreak threshold (0.11 COTS/Tow) (n = 95) at any time were included in the following analyses. This ensures that the comparisons and trends observed are more reflective of reefs where COTS outbreaks pose a threat to coral cover, thus allowing for a more robust assessment of management actions.

To determine how COTS densities changed in response to each management action, the mean number of COTS/Tow was calculated for each reef in each sector during outbreak years. The density of COTS for the 3^rd^ outbreak wave was then compared to the density of COTS for the 4^th^ outbreak wave in every sector using a Bayesian hierarchical model in which the density of COTS was the response variable. Sector (e.g., Cairns and Townsville) and outbreak wave (i.e., 3^rd^ vs 4^th^) were explanatory fixed variables, including their interaction. To account for local variation in COTS densities among reefs, Reef was included as a random effect in the model (for the specific model syntax, see the S2 Table in S1 Appendix). This allowed a test of how the management action applied in each sector affected COTS densities during the 4th outbreak wave.

A second Bayesian hierarchical model was used to assess net changes in coral cover under each management action. We hypothesised that early management intervention would have reduced the amount of coral loss (or resulted in a net gain) compared to the 3^rd^ outbreak. The relative change in coral cover was calculated by comparing estimated individual reef levels of coral cover at the beginning and end of each COTS outbreak wave as defined by Table 1. Change in coral cover was the response variable, and as above, sector and outbreak wave were explanatory fixed variables with the individual reef as a random effect. Both COTS density and coral cover models were fit using and were run for 4000 iterations, with the first 25% of iterations discarded as burn-in (1000 iterations). Three separate chains were run for each model which were thinned at five step intervals. For both models, all chains were well mixed when trace plots were examined and converged on stable posterior distributions (all rhat < 1.01). Goodness-of-fit diagnostics were confirmed using a range of posterior probability checks as well as exploring patterns in simulated residuals.

We also assessed how coral cover changed during the outbreak depending on the management action. For every reef surveyed, the relative change in coral cover between each year was calculated for up to six years following the onset of the 4^th^ outbreak wave. Outbreaks of shorter duration (Cairns, Capricorn Bunker) were modelled for the 4 years of available data. The coral cover trajectories were modelled for each sector individually and with respect to the management action (i.e., Limited, Reactive, Timely, Proactive) applied in that sector. Due to the limited timeframe of the expected outbreak wave in the Cape Upstart sector, the ‘Prevent’ management action was excluded from this analysis (n = 55). The change in coral cover over time was then modelled using generalised additive models (GAM) to accommodate the non-linear nature of the data. Models were fit to each sector and to the aggregated management action. These models were fit assuming a gaussian response term, using a cubic spine smoothing term and maximum of 4 knots. These models track how coral cover changed each year during the current 4^th^ outbreak wave in each sector representing three management actions.

Lastly, to assess the effect of increased levels of culling effort, the annual absolute percent change in coral cover was calculated for each reef during the 4^th^ outbreak wave. This was calculated as the percent coral cover change from the start to the end of the outbreak period divided by the duration in years. Culling effort was defined as the ratio of effort applied and COTS density (culling hours / max COTS density), to determine the median level of cull effort invested in each reef. Using this ratio allows the effort deployed at a reef to be compared relative to COTS density, to account for the increasing levels of effort required at reefs with higher density COTS populations. Reefs were thus categorised as:

- *No COTS Outbreak*: No COTS Outbreaks recorded (<0.1 COTS/tow) to act as a control group where COTS predation is not a major driver of coral mortality
- *Above median culling effort*: Recorded COTS outbreak (>0.1 COTS/tow) AND above median culling effort
- *Below median culling effort*: Recorded COTS outbreak (>0.1 COTS/tow) AND below median culling effort
- *No culling effort*: Recorded COTS outbreak (>0.1 COTS/tow) AND no culling effort

Median culling effort at an individual reef was 121.31 hours per COTS/Tow. Therefore, the level of culling hours needed to be classified as “above median culling effort” for a reef at Potential Outbreak level (0.1 COTS/Tow) was much lower (12.131 hours) than for a Severe Outbreak reef (1 COTS/Tow; 121.31 hours). A Bayesian hierarchical model was constructed to compare the percent annual change among these categories. The model assumed a gaussian response and included sector as a random variable to account for large scale spatial variation.

## 3. Results

### 3.1. Changes in coral cover and COTS densities by management objective and sector

#### 3.1.1 Limited action–Cooktown-Lizard Island and Swain sectors

**Fig 2:**
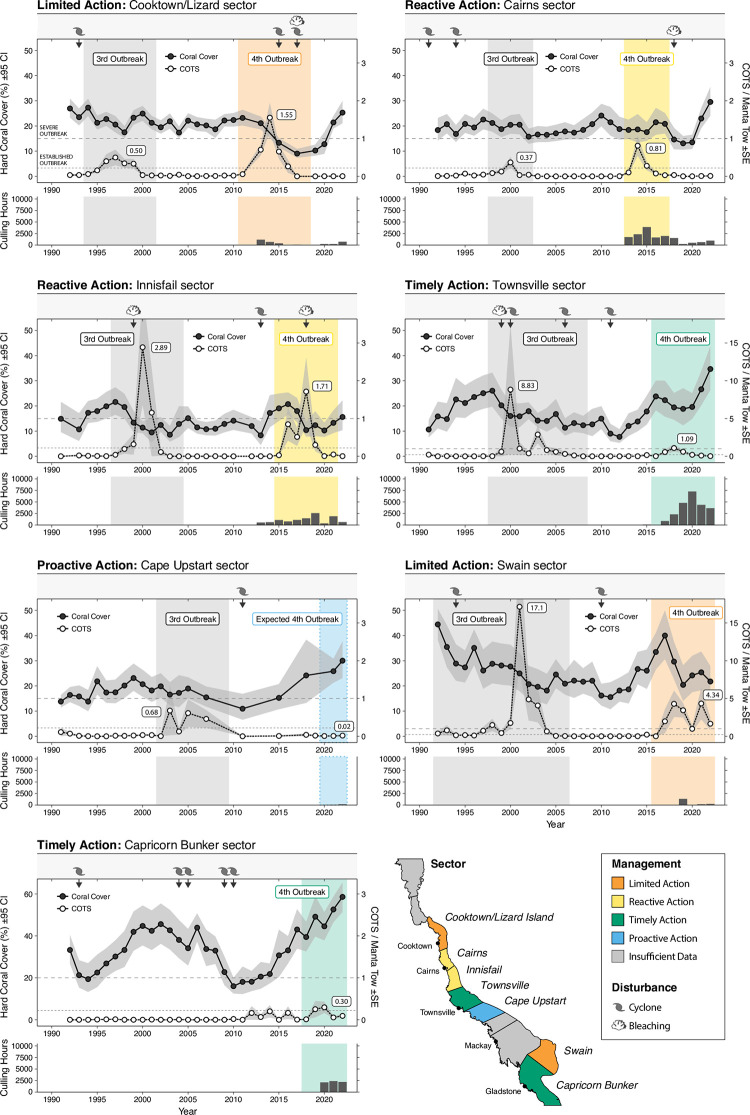
COTS and Coral cover trajectories by sector. Temporal trends in modelled median hard coral cover (black symbols) and raw mean COTS densities (white symbols) from seven focal latitudinal sectors, categorised by the four management Actions (Limited, Reactive, Timely, Proactive) applied during the 4th COTS outbreak. Grey bands are the 95% credible intervals for coral cover and +/- Standard error for COTS densities. Horizontal dashed line is the threshold for a severe outbreak (>1 COTS per tow), while the grey dotted line represents the threshold of an established outbreak. Symbols and arrows along the top of each panel represents the timing of major cyclone and coral bleaching disturbances that resulted in sector-wide mortality. Importantly the plot shows when the mortality was observed which can lag the actual event by up to 1 year. Bar plots below each temporal plot is the total amount of culling effort invested per year. Culling effort represents the remobilised Program from 2012, and site-scale culling during the 3^rd^ Outbreak wave is not displayed. NB the Townsville and Swain Sector plots COTS axes are on a different scale due to higher observed COTS densities. Sector outlines obtained with permission from AIMS under a CC BY license, original copyright 2023.

**Fig 3:**
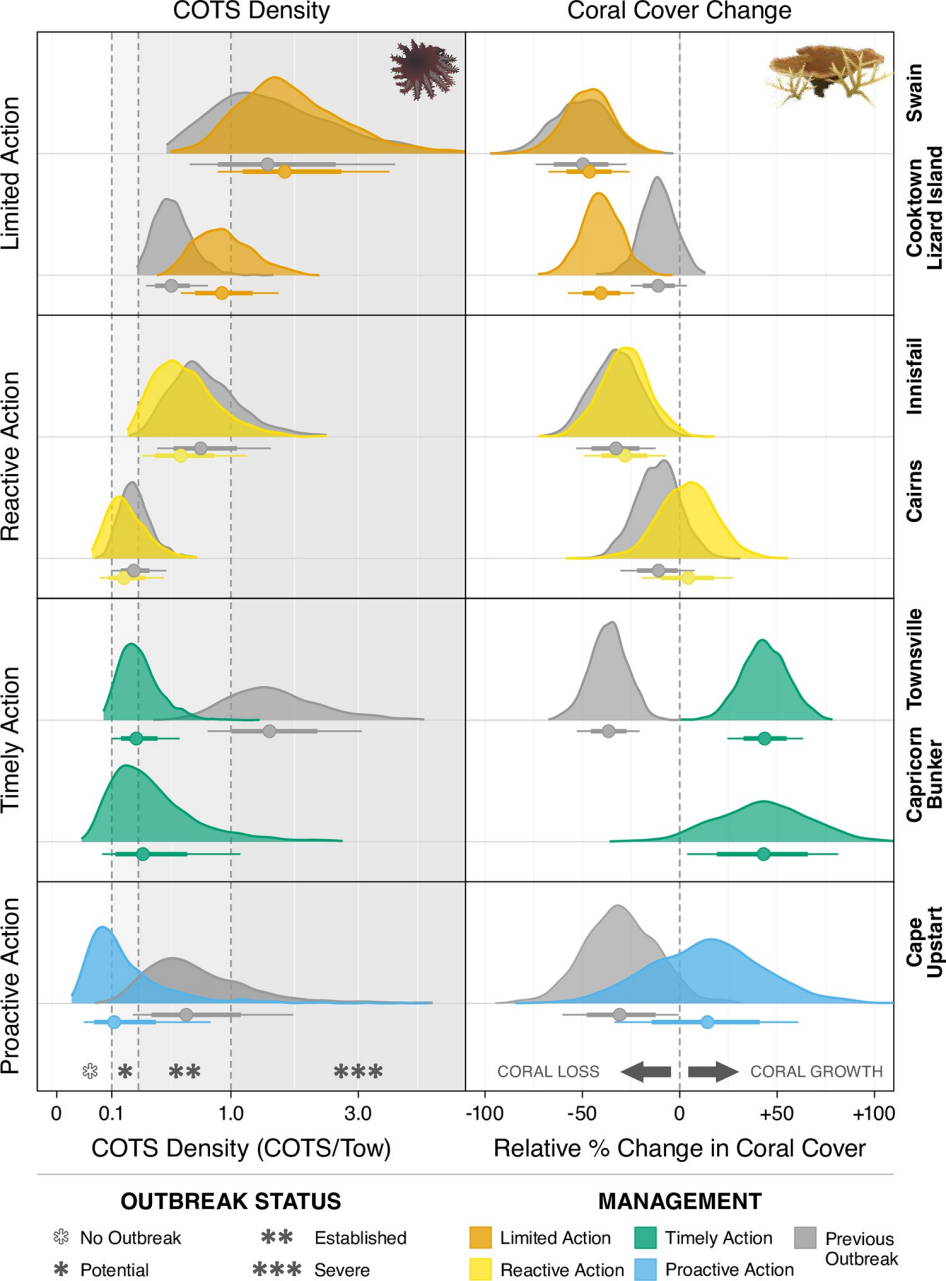
Model outputs comparing outcomes from the 3^rd^ and 4^th^ COTS outbreaks. Posterior probability distributions from Bayesian generalised linear mixed models of: A) Mean COTS densities per manta tow and, B) mean relative change in % coral cover during the previous 3^rd^ (1993; grey) and current 4^th^ (2010; coloured) COTS outbreaks. Data points below probability distributions are the mean responses ±66% (thick bars) and 90% (thin bars) credible intervals. Sectors are colour coded according to the management action: Limited Action (orange; CL = Cooktown-Lizard; SW = Swain), Reactive Action (yellow; CA = Cairns; IN = Innisfail), Timely Action (green; TO = Townsville; CB = Capricorn-Bunkers), and Proactive Action (blue; CU = Cape Upstart). Note that only one posterior probability distribution is shown for the Capricorn Bunker sector as there was no population outbreak in 3^rd^ Outbreak.

**Fig 4:**
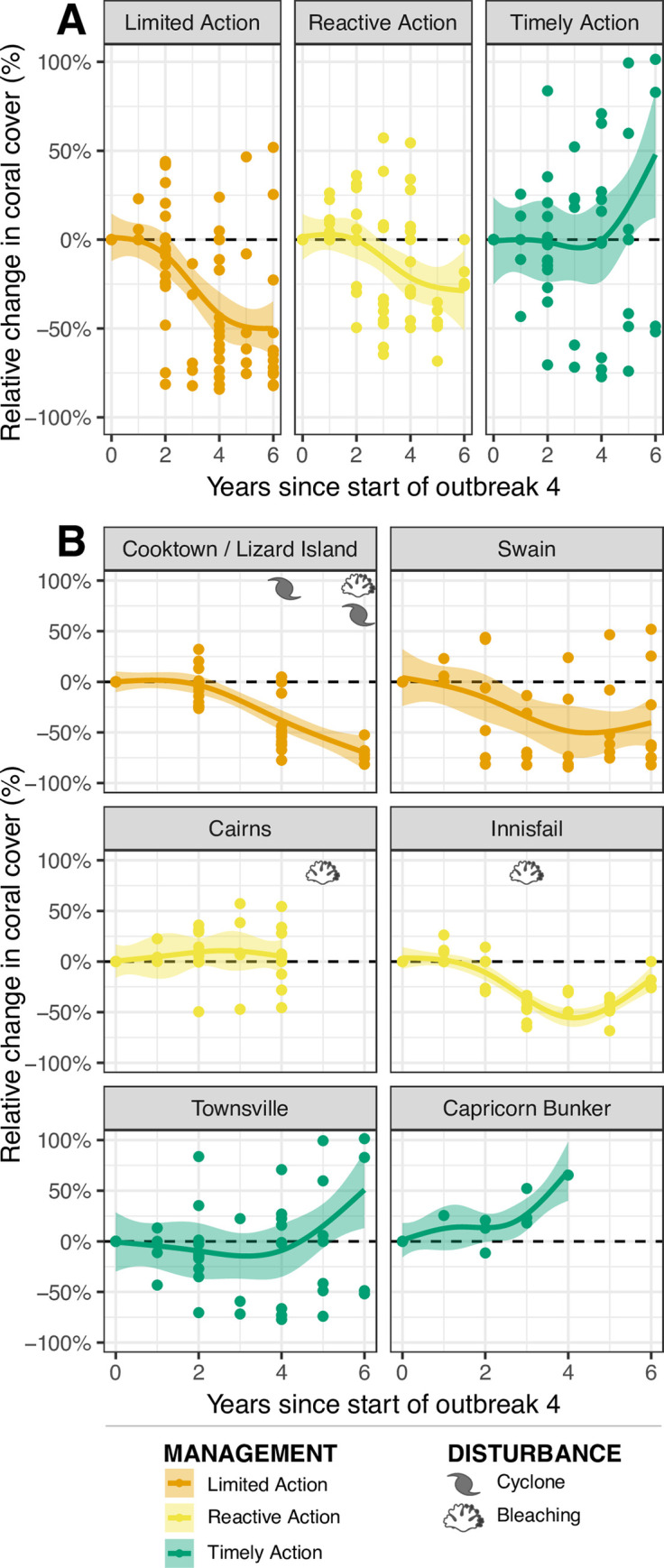
Relative coral cover change during the 4^th^ outbreak. Relative change in coral cover (%) according to the type of management action implemented (A): ‘Limited Action’ (orange), ‘Reactive’ (yellow), and ‘Timely’ (green) and by sector (B). ‘Proactive’ is not shown due to insufficient data for this analysis. Each point represents the relative change in coral cover at a given reef, up to 6 years following the start of the sector-specific 4^th^ outbreak wave (see Table 1). The trendline is fitted using a generalized additive model (GAM) and the transparent ribbon represents the 95% confidence intervals. Disturbance markers indicate timing of events that resulted in sector-wide coral mortality (i.e. not all disturbance events).

The Cooktown-Lizard Island sector in the Northern GBR, or the Swain sector in the Southern had the highest mean COTS densities during the 4^th^ outbreak wave when compared to all other sectors managed by the COTS Program (Fig 2). There was no difference in COTS densities between the 3^rd^ and 4^th^ outbreak waves for the Cooktown-Lizard Island sector in the Northern GBR, or the Swain sector in the Southern GBR (Fig 3, top left). Both sectors had a substantial proportion of their highest posterior density (HPD) interval (90% for the Swain and 40% Cooktown-Lizard Island) above 1 COTS/Tow (Fig 3), indicating a considerable number of reefs in these sectors had ‘severe outbreak’ population levels during the 4^th^ outbreak wave. Indeed, there was a 97% probability that COTS densities were higher in the Cooktown-Lizard Island sector in the 4^th^ outbreak wave (median 0.90 COTS/Tow [0.42: 1.48 90% credible intervals (90% CI)]) than in the 3^rd^ (median 0.43 COTS/Tow [0.23: 0.69 90% CI]). Furthermore, there was 69% probability that COTS densities were higher in the Swain sector in the 4^th^ outbreak wave (median 1.72 COTS/Tow [0.74: 3.34 90% CI]) compared to the 3^rd^ (median 1.45 COTS/Tow [0.39: 3.18 90% CI]) (Fig 4 and S3 Table in S1 Appendix). Limited resources paired with the remote location of these sectors relative to major ports, meant minimal culling effort could be allocated to reefs in these sectors (3,558 (5.7% of total cull effort) culling hours in Cooktown-Lizard Island and 1,599 (2.6%) culling hours in the Swain sector–Table 1).

The limited culling effort correlated with significant and pronounced declines in coral cover in the years following the start of the outbreak in these sectors. The Swain sector experienced severe and temporally consistent declines in coral cover during both the 3^rd^ and the 4^th^ outbreak waves, (3^rd^ = median -50% [-73: -27 90% CI]; 4^th^ = median -46% [-68: -27 90% CI]), with Cyclone Fran (1992) as the only other sector-wide disturbance indicated by the LTMP surveys. As no other major disturbance was recorded, the coral loss observed during the 4^th^ outbreak wave is a primarily a result of COTS predation (S5 Fig). Coral cover decline in the Cooktown-Lizard Island sector was more pronounced during the 4^th^ outbreak wave (median -41% [-58: -25 90% CI]) than during the 3^rd^ (median -11% [-24: 4 90% CI]). The increased coral loss recorded during the 4^th^ outbreak is attributable a more severe disturbance regime during the 4^th^ outbreak wave with (S5 Fig) increased COTS predation, but also the cumulative impacts of two severe tropical cyclones (Ita 2014 and Nathan 2015) and the 2016 and 2017 mass coral bleaching events (S5 Fig) [49,50].

#### 3.1.2 Reactive action–Cairns and Innisfail sectors

Culling effort during the 4^th^ outbreak wave was over four-fold greater in the Cairns sector (15,352 hours (24.8%); Fig 2) and nearly three-fold greater in the Innisfail sector (11,082 hours (17.9%)) than in the Cooktown-Lizard Island and Swain (Limited Action) sectors. Culling resulted in a slight decrease in COTS density between outbreak waves for both Cairns sector (3^rd^ = median 0.20 COTS/Tow [0.08: 0.34 90% CI]; 4^th^ = median 0.14 COTS/Tow [0.04: 0.32 90% CI]), and Innisfail sector (3^rd^ = median 0.69 COTS/Tow [0.24: 1.30 90% CI]; 4^th^ = median 0.50 COTS/Tow [0.18: 1.02 90% CI]) although, statistically, these densities were similar (Fig 4). Culling only commenced in these sectors when densities had already reached ‘potential outbreak’ levels (Fig 2: 0.1–0.22 COTS/tow). The limited resources available and the rapid advancement of the outbreak meant that COTS densities could not be effectively suppressed to sustainable levels across these sectors.

However, culling was focused on priority reefs; chosen based on their significant ecological and/or economic value. Indeed, this focused effort resulted in effective mitigation of the coral declines expected in the absence of culling. Culling effort during the 4^th^ outbreak in the Cairns sector corresponded with a reverse in the mean relative change in coral cover from a decline during the 3^rd^ outbreak (median -11% [-29: 8 90% CI]) to no change during the 4^th^ outbreak (+4% [-18: 28 90% CI]), and although both outbreaks credible intervals intersect zero (Fig 3), the probability of hard coral cover declining was 85% and 39% for the 3^rd^ and 4^th^ outbreak waves respectively. The disturbance regime (of non-COTS disturbances) was broadly similar in the 3^rd^ and 4^th^ outbreak waves with increased mortality from coral bleaching offset by reduced mortality from storms during the 4^th^ outbreak wave. Coral loss attributed to COTS was approximately halved, indicating the change in coral outcomes was primarily related to COTS predation (S5 Fig). Importantly, the observed net-zero change in coral cover during the 4^th^ outbreak wave occurred prior to the widespread coral mortality attributed to the 2016 and 2017 mass bleaching events (N.B. Mortality is often observed in the year following the event and thus these events appear as 2017 and 2018 on Figs 2 and S5). Conversely, in the Innisfail sector, the 4^th^ outbreak window coincided with the bleaching event (Figs 2 and S5) and thus any coral protection was offset by bleaching and coral outcomes were similarly negative for both the 3^rd^ (median -33% [-53: -12 90% CI]) and 4^th^ (median -28% [-49: -7 90% CI]) outbreak waves (Fig 3).

#### 3.1.3 Timely action–Townsville and Capricorn Bunker sectors

Timely and adequate intervention effectively suppressed COTS densities in the Townsville and Capricorn Bunker sectors (Fig 3). In the Townsville sector, there was a significant, 7-fold decrease in the density of COTS between successive outbreak waves, from a median of 1.50 COTS/Tow [0.52: 2.68 90% CI] during the 3^rd^ outbreak wave to a median of only 0.21 [0.07: 0.41 90% CI] COTS/Tow during the 4^th^ (Fig 3). Timely, sufficient, and sustained culling effort (23,534 hours (37.9% of total effort)) alongside implementation of the IPM framework, prevented a reoccurrence of the ‘severe outbreak’ scenario observed during the 3^rd^ outbreak wave despite sufficient hard coral prey to sustain a severe outbreak. Crucially, culling commenced on Townsville sector reefs in 2015, which coincided with sector-wide COTS density exceeding the 0.11 COTS/Tow ‘potential outbreak’ threshold (Fig 3). Additionally, it is likely that there was some benefit derived from the upstream culling conducted in the Cairns and Innisfail sectors. Despite not having a prior outbreak wave for comparison in the Capricorn Bunker sector, COTS densities have been effectively suppressed throughout the 4^th^ outbreak with timely effort (6,619 hours (10.7%)), with the median 0.25 COTS/Tow [0.03: 0.83 90% CI] sitting just above the lower threshold of an ‘established’ outbreak (Fig 3). Less effort was required to effectively manage COTS outbreaks in the Capricorn Bunker sector to due to there being fewer reefs in the sector, earlier intervention and lower overall COTS densities.

The benefit of this management action becomes starkly apparent when assessing the relative change in coral cover. The 3^rd^ outbreak wave in the Townsville sector correlated with a significant mean relative decline in mean coral cover of -37% (-51: -20 90% CI; Fig 4). In contrast, there was a significant relative increase in coral cover during the 4^th^ outbreak wave of 44% (23: 62 90% CI; Fig 4). The credible intervals surrounding both outbreak estimates are narrow and do not overlap zero, adding further support to the clear difference in coral cover outcomes between the 3^rd^ and 4^th^ outbreak waves. In the Capricorn Bunker sector, a relative increase in mean coral cover of 43% (4: 81 90% CI) was recorded, although the variance spanned a larger range of values. The 2017 bleaching event contributed to the initial decline in the Townsville sector at the onset of the outbreak which was mirrored by a similar magnitude bleaching event at the start of the 3^rd^ outbreak (S5 Fig). While this was not explicitly modelled in this study this indicates that our findings of sector-level coral protection in the Townsville as a direct result of COTS culling is supported by investigating the full disturbance context. The Capricorn Bunker sector had some minimal coral mortality attributed to bleaching during the 4^th^ outbreak window but was essentially disturbance-free (S5 Fig). This difference in disturbance exposure combined with the earlier intervention may partially explain the overall higher levels of coral found in the Capricorn Bunker sector.

#### 3.1.4 Proactive action–Cape Upstart sector

Mean COTS densities in the Cape Upstart sector (to the south of Townsville) suggest a decrease from the 3^rd^ outbreak (median 0.56 COTS/Tow [0.09: 1.45 90% CI]) to the 4^th^ outbreak (median 0.11 COTS/Tow [0.01: 0.50 90% CI]) (Fig 3). While the uncertainty estimates are large in this sector due to the small sample sizes, it appears that mean COTS densities during the 4^th^ outbreak wave were the lowest of all sectors, despite low levels of culling effort being allocated to reefs in this sector (221 hours (0.4%). This result indicates that successful suppression in upstream sectors (Townsville) may have delayed the onset of the outbreak and thus fewer culling resources were required. A relative increase in coral cover of 14% (-33: 61 90% CI) during the 4^th^ outbreak was recorded, noting that the uncertainty estimates are large and intersect zero. However, the majority (68%) of the posterior probability distribution was above 0, suggesting that it was more likely that coral cover increased than decreased during the 4^th^ outbreak wave. In contrast, there was a relative median decline in coral cover of -31% (-60: -1 90% CI) during the 3^rd^ outbreak. During the 3^rd^ outbreak wave Cape Upstart also recorded coral mortality attributed to storms and thus the comparative increases in coral cover during the 4^th^ are a result in both the reduction in COTS densities (primarily from upstream culling) and decreased exposure to storms (S5 Fig). Importantly however the coral loss attributed to storms was roughly half that of COTS during the 3^rd^ outbreak wave indicating that the differences in coral outcomes are still primarily driven by COTS.

### 3.2. Temporal changes in coral cover during the 4^th^ outbreak

The relative change in coral cover (six years following the onset of the 4^th^ outbreak wave) varied greatly among regions, partly reflecting the timing and magnitude of culling effort as well as concurrent coral mortality events. Reefs within sectors that received ‘Limited Action’ (Cooktown-Lizard Island and Swain) were subject to substantial decreases in coral cover over the course of the outbreak wave (Fig 4). Reef-wide estimates of coral cover decline (approximately 50%) peaked in the fifth year following the onset of the 4^th^ outbreak wave (Fig 4). Importantly, the Cooktown/Lizard Island sector was also heavily impacted by cyclones (2014/15) and bleaching (2016/17) resulting in a more uniform decline in coral cover compared to the Swain sector which had limited exposure to other disturbances, resulting in coral growth on some reefs (S4 Fig). Sectors with ‘Reactive Action’ (Cairns and Innisfail) generally retained coral cover during the initial few years of an outbreak (Fig 4B). However, in both sectors, adequate culling effort was not able to be sustained and coral bleaching events in 2016 and 2017 ensured coral cover declined over subsequent years of the outbreak (~25% decline after six years).

**Fig 5:**
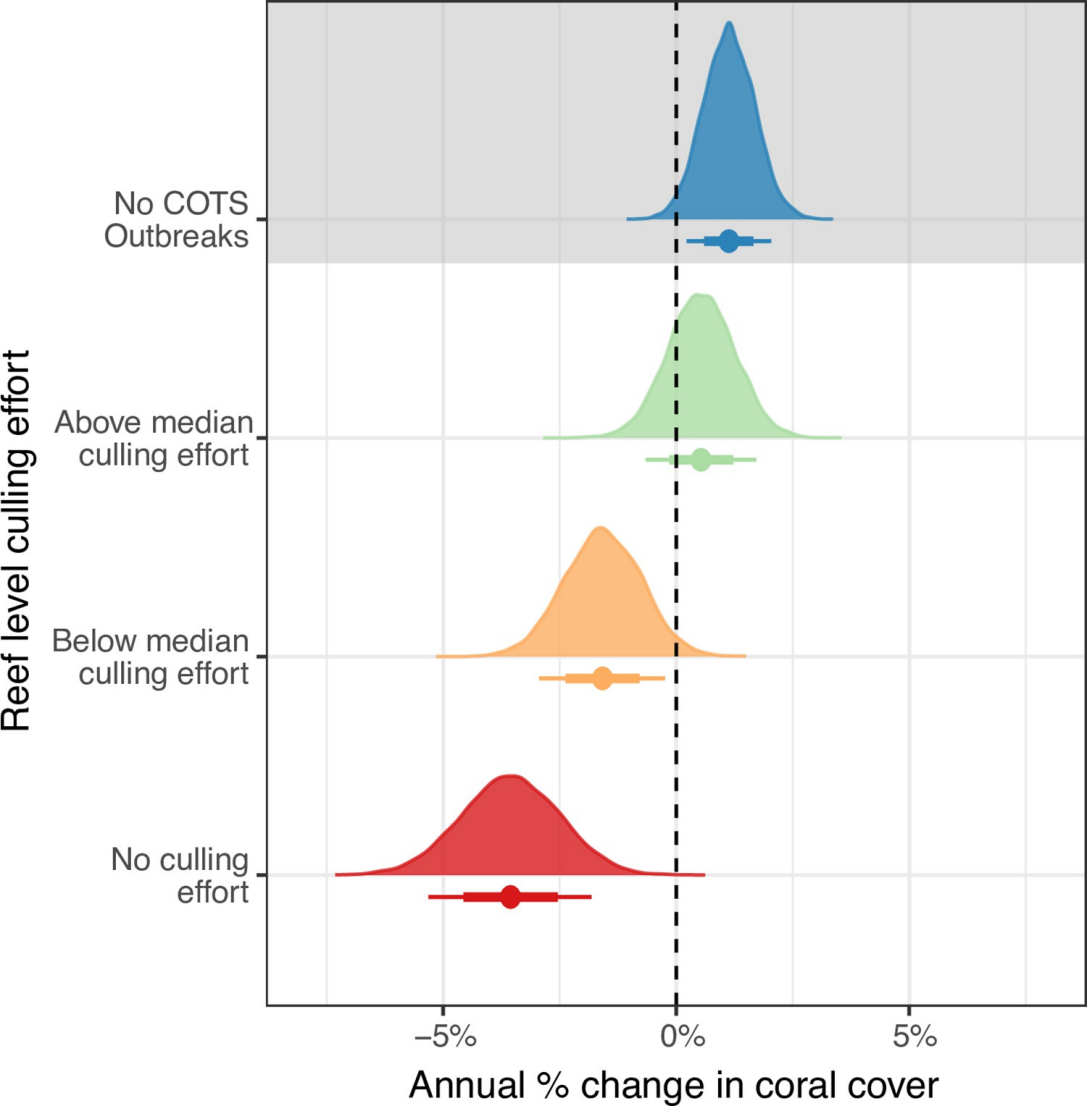
Annual coral cover change at varying intensities of COTS control. Comparison of posterior probability distributions from Bayesian generalised linear mixed models of annual change in coral cover at increasing levels of culling effort observed during the 4^th^ outbreak wave. Data points below probability distributions are the mean responses ±66% (thick bars) and 90% (thin bars) credible intervals. Culling effort is categorised based on the number of culling hours divided by the maximum COTS density observed during the 4^th^ outbreak wave (see Table 1 for time period), as (1) reefs with no recorded COTS outbreak (blue); (2) reefs with recorded COTS outbreaks and above median culling effort relative to maximum COTS density (green); (3) reefs with recorded COTS outbreaks and below median culling effort relative to maximum COTS density (orange) and (4) reefs with recorded COTS outbreaks and no culling effort (red).

Coral cover trajectories on reefs within ‘Timely Action’ sectors (Townsville and Capricorn Bunker) were substantially different than those in the ‘Limited Action’ and ‘Reactive’ sectors. Initially, coral cover on ‘Timely’ reefs is maintained; neither increasing nor decreasing (Fig 5). However, by the 4th year, there is a rapid positive increase in coral cover, demonstrating the benefits of timely and consistent intervention during low disturbance periods. Six years after the start of the outbreak, coral cover is estimated to have increased by approximately 50% across sectors where COTS outbreaks were effectively suppressed (Timely Action). Importantly, as these outcomes are defined coarsely at the sector-wide scale there are outliers within each category. For example, two reefs within ‘limited action’ recorded significant relative increase in coral cover (~40%), due to COTS densities not reaching outbreak densities, and the absence of other major disturbances.

Variability in the coral cover trajectories of outlier reefs led to increased uncertainty of the sector-wide trajectories (Figs 5 and S4). In the Townsville sector, while the management action was considered ‘timely’ at a sector-wide level, three reefs (John Brewer, Little Kelso and Rib; S4 Fig) more closely resembled ‘Reactive’ management action where COTS control was initiated after the Severe Outbreak threshold had already been exceeded. Delayed initiation of control effort on these reefs resulted in greater coral loss and a slower recovery trajectory compared to other ‘Timely Action’ reefs. Similarly, the trajectory of Mackay Reef differs from the rest of the Cairns sector (Reactive Action) due to the earlier onset and higher severity of the COTS outbreak (S4 Fig, AIMS Reef Dashboard: https://apps.aims.gov.au/reef-monitoring/reef/mackay%20reef/manta). Additional coral losses from bleaching and cyclone disturbances also contributed to the observed temporal trajectories. Cumulative impacts were exemplified at St Crispin Reef (Cairns sector; Reactive Action), which was heavily impacted by the 2016 bleaching event, negatively affecting the coral recovery trajectory of the reef, while Green Island and Hastings Reefs showed strong coral growth due to high levels of timely COTS control effort (>800 hours) (S4 Fig). Conversely, in the Swain Sector (Limited Action), East Cay Reef and Unnamed Reef 21–558 experienced large increases in coral cover, as these reefs had been recovering from previous cyclone damage (S5 Fig) and had not yet been exposed to COTS outbreaks. Importantly, all the trends observed within sectors and among management action categories are clearly defined even when these outlier trajectories are included in the analyses and would be strengthened by excluding them (Figs 4 and S5). This closer examination of outliers further reinforces reinforce the importance of timely and sustained COTS control to improve coral cover outcomes as well as the necessity of understanding the broader disturbance regime to interpret broad-scale patterns in coral cover (S5 Fig).

### 3.3. Culling effort and coral cover dynamics

Irrespective of geographical distribution, reefs with no detected COTS outbreak (<0.1 COTS/Tow) had an estimated 1.1% [0.2%: 2.0% 90% CI] annual increase in coral cover, indicating net coral growth in the absence of COTS predation (Fig 5). Importantly, coral cover increased by 0.54% per year [-0.6%: 1.7% 90% CI] on reefs with COTS outbreaks that received above median levels of culling effort. This corresponded to approximately half (49%) of the estimated annual increase in coral cover at non-outbreak reefs.

Conversely, on reefs where COTS outbreaks were detected and no culling effort was deployed, coral cover declined by -3.6% [-5.4%: -1.8% 90% CI] annually. Although outbreaking reefs that received below median levels of culling effort experienced net coral loss, the rate of coral cover decline was reduced by 56% (-1.6% [-3.0%: -0.26% 90% CI] per year) relative to outbreaking reefs that did not receive culling effort.

## 4. Discussion

### 4.1. Overall

Differences in suppression of COTS outbreaks and coral cover protection depended on the timing of management action, level of effort invested and the frequency and severity of concurrent disturbance events. Comparing the outcomes of the 3^rd^ and 4^th^ COTS outbreaks (Fig 3) and coral recovery trends, our results indicate five key findings. Firstly, where timely management and sustained effort was applied, the extent of coral loss was substantially reduced, and COTS outbreaks were suppressed protecting and contributing to rapid coral recovery from COTS predation and other disturbances (Townsville, Capricorn Bunker sectors). Secondly, where COTS management is reactive, significant culling effort must be applied to reduce COTS densities, restricting the outcome to coral cover maintenance and/or a reduction in the extent of loss (Cairns and Innisfail sectors). Thirdly, there is also evidence that proactive effort combined with timely intervention in upstream areas can mitigate the southward spread of the outbreak wave (Cape Upstart sector). Fourthly, where limited or no action was applied, COTS densities reached severe levels causing extensive coral loss. At the reef level, our findings clearly show that increasing levels of culling effort resulted in substantial increases in annual coral growth, particularly in the absence of other major disturbance events. Indeed, reefs above median culling effort retained almost half of the coral gain recorded at non outbreak reefs (Fig 5). Importantly, other impacts to coral during the course of management action interact with outcomes potentially offsetting coral cover gains (i.e. Cairns, Innisfail sectors) and must be carefully considered when interpreting these results (S5 Fig). With respect to the Townsville sector however, where the largest positive impact of culling is posited, the disturbance history of both outbreak periods was similar and primarily attributed to COTS (~60–70% S5 Fig). While this was not explicitly modelled in this study this indicates that our findings of sector-level coral protection in the Townsville sector as a direct result of COTS culling is supported by investigating the full disturbance context. These findings represent a best-case scenario of what can be achieved during a low disturbance window by limiting the mortality associated with COTS.

Our findings suggest that COTS control may substantially improve coral growth trajectories for reefs where timely, sustained control effort is sufficient to suppress or prevent COTS outbreaks. The observed 1.1% y^-1^ increase in coral cover on reefs with no detected COTS outbreak (densities below 0.1 COTS per manta tow) provides the first empirical evidence to support the De’ath et al. 2012 [13] projection that coral cover on the GBR would have increased by 0.89% y^-1^ in the absence of COTS predation. Most importantly, our results indicate that at reefs with significant COTS outbreaks and effective culling effort, coral growth was maintained at 0.54% y^-1^ during the 4^th^ outbreak wave. These results also show that while less effective, even below median levels of culling effort reduce coral losses by 2% y^-1^ (from 3.6% y^-1^ to 1.6% y^-1^) compared to no culling. Combined, these results highlight that sustained, sufficient COTS control protects coral growth (or at least reduces coral losses) even in the presence of other concurrent reef health impacts.

Our findings of regional scale outcomes align with recent modelling that a fleet of 10–15 vessels could deliver significant regional COTS outbreak suppression and coral growth and recovery effects under projected climate futures [51–53]. Our analyses estimate increases in coral cover consistent with previous predictions [13] in sectors where COTS have been effectively managed (e.g. Townsville, Capricorn Bunker, and Cape Upstart sectors; Figs 3 and 4) and at reefs with above median culling effort relative to COTS density (Fig 5). Although these estimates are a simplification of a large and complex system, our findings demonstrate that regional-scale COTS control protects coral growth, promotes recovery from cumulative impacts, and consequently increases the resilience of the GBR.

### 4.2. COTS suppression and coral protection outcomes

Coral outcomes at the sector level were primarily driven by the management action applied (i.e. the timeliness of action) and the exposure to disturbance events. Reefs in sectors with ‘Timely Action’ had an approximate 50% relative increase in coral cover over the 6-year time horizon from the initiation of sector-wide outbreaks. These results show marked differences to reefs with ‘Limited Action’ (approximately 50% coral loss) or where reefs were managed reactively (‘Reactive Action’, approximately 25% coral loss; Fig 4). These results clearly indicate significant improvement in coral outcomes associated with early intervention in the management of COTS populations [54]. Additionally, reefs with higher levels of culling effort experienced reduced levels of coral loss and increased gains in coral cover (Fig 5). While COTS control has been shown to effectively protect coral cover at the site scales [37,55], this research provides the first published empirical evidence for reef and sector-level COTS outbreak suppression and coral protection. These findings support, and in some cases exceed, the estimated regional benefits of targeted COTS control [51–53] verify the benefit of early and sustained culling effort at large spatial scales.

Even sectors with reactive management actions, once outbreaks were established, were afforded some protection from COTS. Although the culling effort in these sectors did not lead to significant decreases in COTS densities, it did manage to preserve coral cover, and certain ecological processes (e.g. coral larval export and connectivity) on select, target reefs (e.g. Green Island, Hastings Reef, S4 Fig) that are disproportionately important for tourism. Moreover, the reef level analysis (Fig 5) revealed that even below median culling effort afforded coral protection (compared to no culling) on reefs were sustained resources were not available. This effect is typified by reefs in the Cairns sector (Fig 4) which showed a net-zero change in sector-wide coral cover prior to the 2016 and 2017 mass bleaching events [50,56]. Counterintuitively, a net-zero change in coral cover represents possibly the best-case scenario for reactively managed sectors. By definition, the culling effort applied late in this sector’s outbreak cycle means that culling effort was focussed on select reefs of high ecological and economic value. The preservation of adult corals on important coral source reefs likely provided this sector with adequate larval supply mitigating losses caused by COTS [57]. Cairns sector reefs have some of the greatest economic and recreational value on the entire GBR due to their proximity to major urban centres and focus of tourism [39]. Therefore, by investing moderate amounts of culling effort even after the outbreak was established, reefs critically important for recreation and tourism were protected, and the overall downstream supply of COTS larvae was reduced [28].

### 4.3. Compounding benefits offset cumulative impacts

A key finding is the apparent accrual of compounding benefits as the outbreak progressed and culling effort was sustained. Compounding benefits refers to the proposed pathway though which the numbers of COTS larvae are reduced, and coral larvae are increased over successive years as more outbreaks are suppressed and coral is protected at upstream reefs and sectors. Recent, GBR-wide modelling of coral and COTS larval connectivity has enabled the identification of high priority reefs that may disproportionately drive coral and COTS population dynamics [28,29]. By targeting COTS control to these priority reefs that are important sources of coral and COTS larvae, the benefits of culling should begin to compound across reefs and sectors. In the early stages of the COTS Program (2012–2015), most resources were spent controlling Established or Severe outbreaks on reefs with high tourism value. Despite the limited resources, reactive action was still able to deliver some coral protection benefits, particularly within the Cairns region, however the localised benefits of this strategy were limited by the 2016 and 2017 mass bleaching events [50,56](Fig 3). Nevertheless, the continued removal of adult COTS and the protection of coral in upstream sectors (i.e. Innisfail, Cairns) may have impacted the Townsville sector, amplifying the benefits of intensive culling in the region. Importantly, this highlights the need to ensure that the coral benefits (and COTS larval suppression) from COTS control are accrued as early as possible within the outbreak cycle, allowing them to compound more rapidly during low disturbance windows. Put simply, sustained culling delivers compounding sector scale benefits.

During the 2018–2022 period, no significant mortality event was recorded in the GBR despite two mass bleaching events in 2020 and 2022 (Great Barrier Reef Marine Park Authority 2020; 2022). This period of low relative coral mortality coincided with a time where COTS were effectively suppressed in the Townsville sector resulting in a delayed proliferation to the neighbouring Cape Upstart sector. It is important to note that the reduced sample size and limited culling activity in Cape Upstart makes it difficult to attribute positive coral outcomes directly to culling. More accurately, they appear to be a product of the culling in upstream sectors and the reduced disturbance regime during the 4^th^ outbreak wave. By maintaining much higher levels of coral cover during the recent outbreak wave compared to the 3^rd^, COTS control appears to have aided the rapid recovery of coral cover to historic highs in both sectors during a limited disturbance period (Fig 3) [58].

### 4.4. COTS Control as a tool for resilience-based management

Resilience-based management (RBM) focuses on improving ecosystem resilience both by mitigating coral loss and aiding recovery. While the risk from stressors such as marine heat waves from climate change require global action to reduce greenhouse gases, local management actions such as COTS control can play an integral role in RBM on the GBR [34]. COTS control enhances the resilience potential by mitigating coral loss from starfish predation which increases coral larval supply and recovery [29]. COTS control also diminishes the reproductive potential of the COTS populations, and thus coral loss at downstream reefs [17,28,36,59]. This, in turn, allows the reef system to capitalise on low disturbance periods and recover more rapidly. Our findings suggest that during low climate disturbance periods reefs on the GBR can recover rapidly from COTS outbreaks if coral loss is minimised and coral cover is maintained above 10–20% (Fig 3, Townsville Sector). These findings align with previous “resilience threshold” estimates of approximately 17% coral cover; above which positive coral accretion rates are maintained [60–62] causing coral cover to increase exponentially [4,63]. Maintaining coral cover above these levels also ensures that losses from COTS are predominantly limited to the fast-growing preferred coral prey species (e.g., *Acropora* spp), thus protecting the slower growing species typically consumed at the termination of the outbreak [14,18]. By “defending the resilience threshold”, COTS control promotes rapid recovery and prevents the loss of slower growing corals and may thus protect coral diversity at a reef [64].

As the only major driver of coral mortality on the GBR that is amenable to direct local action [14], COTS control currently provides the only effective tool for coral preservation deployable at scale [37,65]. With the predicted increase in frequency and severity of coral bleaching [66,67] and ongoing severe tropical cyclones [68,69], the persistence of a coral dominated GBR will crucially rely on offsetting mortality from these and other disturbance events when possible. While emerging methodologies for coral restoration [70] assisted evolution [71,72] are encouraging, they remain cost prohibitive at large scales [73], and the most effective way to protect corals is to, quite simply, prevent mortality in the first place [74]. Mitigating the coral loss associated with COTS outbreaks promotes the natural recovery of affected reefs which allows restoration efforts to be more effectively targeted towards reefs where coral decline is associated with other disturbances (i.e. bleaching, cyclones). Importantly, as coral reefs are increasingly exposed to extreme thermal stress events, it becomes even more important to ensure the colonies which have survived and thus might be thermally tolerant [75,76] are protected from outbreaks of coral-eating starfish to promote the natural proliferation of these adaptations.

### 4.5. Considerations

The impacts of COTS on coral and the benefits of culling cannot be entirely disentangled from other disturbances [1,50,77]. Declines in coral cover due to COTS may be further exacerbated by thermal coral bleaching and cyclones. Indeed, some of the gains in coral cover observed in the Cairns sector were offset by the 2016 and 2017 coral bleaching events. Therefore, it’s important to consider that the long-term regional benefits of culling to protect coral cover cannot be guaranteed, with increasing frequency and severity of coral bleaching and continued exposure to severe cyclones predicted over the coming decades [78,79]. It is also important to note that the gains in coral cover observed in the Townsville and Capricorn Bunker sectors coincided with a period of limited coral mortality events. Mass bleaching events occurred in 2020 and 2022, however, neither sector experienced widespread mortality events. These events may have halted coral recovery [80], although the full extent of the 2022 event will not be clear until 2023 surveys are completed. While this means that the coral cover benefits shown herein cannot be simply extrapolated into the future, this window of limited disturbance for some sectors has provided the opportunity to assess the impact of COTS control in relative isolation and can act as guide for the potential benefit that could be expected in the absence of widespread mortality events [13]. Crucially however, the disturbance history of the Townsville sector for the 3^rd^ and 4^th^ outbreak waves were comparable (S5 Fig), with most of the coral loss attributed to COTS during both waves (~60–70%). This additional context provides further evidence that the major difference between the coral outcomes for these periods was not driven by other disturbance events but was most likely due to the reduction of the impact of COTS via manual control.

Although there have been four documented COTS outbreaks since the 1960’s [14], LTMP surveys only capture the dynamics of the 3rd and 4th outbreaks meaning only these two could be analysed and compared herein. While some level of natural variation in outbreaks is likely due to stochastic biotic and abiotic factors (e.g. ENSO cycles, chlorophyll-a levels, connectivity patterns) [19,21,81–83], peak densities of COTS were two to three times higher during the 4^th^ outbreak wave in the Cairns and Cooktown-Lizard Island sectors respectively (Fig 3). It seems reasonable to assume that the downstream progression of the 4^th^ outbreak wave, to the Townsville and Cape Upstart sectors, would have been on a very similar trajectory prior to intervention. However, peak densities in the Townsville sector were instead nine times lower during the 4^th^ outbreak wave, suggesting that timely and sustained COTS control effectively suppressed COTS densities in this sector. It is also possible that major disturbance events, like the 2016 and 2017 bleaching and/or Cyclone Debbie (2017), slowed the downstream progression of the outbreak wave by, for instance, reducing the coral prey available for COTS which is key for their recruitment and reproductive success [84,85]. Interestingly, the population replenishment of COTS in the Townsville sector seems, however, not to have been impeded during the 3^rd^ outbreak wave by the 1998 bleaching event (Fig 3) that mostly affected inshore reefs of the GBR. Even though the limited timeframe of the present study constrains our ability to interpret the results, proactive culling during the non-outbreak phase in the Cooktown-Lizard Island and Cairns sectors appears, at least so far, to have maintained COTS densities below outbreak levels (Fig 3). Despite the non-outbreak culling effort, increasing numbers have been observed during fine-scale monitoring near Lizard Island [86] indicating the emergence of a COTS outbreak wave.

It is also important to recognise that the outbreak dynamics in the Capricorn Bunker and Swain sectors behave differently than the reefs/sectors in the central section of the GBR [87]. In the Capricorn Bunker, a sector-wide outbreak was only observed during the 4^th^ outbreak wave, commencing in 2018, but none were recorded during the 3^rd^. Prevailing hydrodynamic patterns likely contribute to these sectors possessing their own outbreak dynamics [21,87,88]. Management of these areas may therefore need to take a more tailored approach that better suites their specific case.

### 4.6. Implications for the strategic management of COTS outbreaks

The coral protection and COTS suppression outcomes provide key insights for the strategic management of COTS on the GBR. Timing and resourcing constraints during the 4^th^ outbreak wave meant that after an initial attempt to suppress the primary outbreak, effort was refocused on action to ‘Protect’ individual high value reefs (S1 Fig). The efficacy of this approach was determined primarily by how early the intervention could start, resource availability and the severity of external, unmitigable disturbance events (i.e. coral bleaching). When effort was applied early with sustained resourcing (i.e. Timely Action: Townsville, Capricorn Bunker) pronounced coral protection and COTS suppression was observed at regional scales. These findings indicate that the higher-level strategic objective of “suppress” is achievable with early intervention and adequate resourcing. Indeed, the Program is actively incorporating emerging research on early detection methods such as eDNA and scooter-assisted large area diver-based (SALAD) surveys to reduce ensure outbreaks are identified and controlled in a proactive or timely manner.

A key learning from the past decade is that while it was logical to prioritise Prevention as the highest order objective, in reality, the higher order outcomes actually accrue from targeted protection of individual reefs. Targeted protection and concentrated culling effort on important source and sink reefs enabled sector-level suppression and arguably a degree of prevention in the progression of the outbreak wave (e.g. Cape Upstart). Thus, the hierarchy of objectives are perhaps better characterised as compounding benefits where targeted and proactive/timely “Protection” enables regional “Suppression” in turn helping to “Prevent” or delay the progression of outbreak waves. The strategic management of COTS should also integrate with other available management actions that can influence COTS outbreak dynamics such as Marine Park zoning [15,89] and water quality improvements [90,91]. Moreover, setting these revised management objectives at a sector level (rather than an outbreak wave) and explicitly incorporating the timeliness of action will provide a more comprehensive approach to COTS management on the GBR.

### 4.7. Suppressing future outbreak waves

Given the significant reduction in COTS densities observed where timely and sufficient culling effort was deployed (i.e. in the Townsville sector), it is evidently feasible to supress future outbreak waves via targeted manual control [54]. Based on the frequency of previous outbreak cycles, the next outbreak wave was forecast to initiate by 2025/26 [54]. Continuous funding of the Program has meant that significant amounts of early surveillance has been applied within the initiation zone in addition to routine monitoring conducted by AIMS. This surveillance detected increasing COTS densities throughout 2021/22 in key areas of the initiation zone (Lizard Island, Eyrie Reef, Batt Reef) and additional fine scale data indicated that the build-up of COTS in the initiation zone was underway [86]. Based on this information, additional culling capacity was deployed to proactively suppress these pre-outbreak populations.

Importantly, mounting evidence suggests that other regions of the Marine Park, such as the Swain [83], Cape Grenville [86] and Townsville/Innisfail sectors [83,92] are particularly susceptible to COTS outbreaks and may play crucial roles in either the overall outbreak wave dynamics or may initiate independently (e.g. Swain sector). Continued research must look to identify clusters of reefs that contribute to the regional initiation and/or amplification of outbreak waves. It is imperative that these important clusters of reefs are routinely monitored to detect emerging outbreaks and sufficient resources are available to suppress them where logistically feasible. Despite the likely lower numbers of COTS which may be culled by implementing proactive or timely action in these regions, early detection and culling intervention will ensure the most efficient use of available resources, and the greatest coral protection outcomes are achieved at reef and regional scales.

## 5. Conclusions

The demonstrated effectiveness of the Program in protecting coral at reef and regional scales (i.e. sectors) solidifies it as an effective tool to enhance Reef resilience. The effectiveness and efficiency of the Program will continue to increase if there is sustained investment in expanded control resources and research to develop new tools for monitoring [93,94] and culling [95], and our knowledge of COTS, their ecology, and outbreak dynamics improves [16]. Recent, long-term funding commitments by the Australian Government will allow the Program to continue operations through to the end of the decade. This is essential to maintain the gains made to date in tackling the 4^th^ outbreak wave and to minimise future outbreak waves (i.e. stop them getting out of control [54]. From a cumulative impact perspective, even though “prevention” of outbreak waves may not ultimately be possible, suppressing the peaks of COTS outbreaks through sustained proactive and/or timely action not only reduces coral predation and outbreak progression, it also avoids the increasingly likely scenario that the peak COTS outbreak losses coincide with other disturbance events leading to unwanted, synergistic negative impacts.

Effective conservation management in the Anthropocene requires a “resilience-based management” approach to develop and implement proactive, direct interventions that protect ecosystem resilience at a time of unprecedented environmental change. Strategically targeted COTS control, at logistically feasible locations, has emerged as a core management tool on the Great Barrier Reef due to its capacity to directly reduce coral mortality at broad scales and minimise the risk that COTS predation will compound coral losses from coral bleaching events and tropical cyclones as well as maximise the coral gains during disturbance free periods. Sustained, sufficient COTS control complements and enhances existing measures including spatial and species management, protecting both natural adaptation and ecosystem resilience under a rapidly changing climate.

## Supporting information

S1 Appendix Supplemental text and tables. Detailed explanations of the use of disturbance data, COTS control program methods, definition of sector-wide management action and the rationale for categorising the Cape Upstart sector as ‘Proactive action’. Also includes supplemental tables: **Table S1. Glossary of key words and acronyms; Table S2.** Model formulae and the description of the variables analysed; Table S3. Summary statistics for COTS densities and Coral Cover for each outbreak by sector.
(DOCX)

S2 Appendix Data and R code for analysis. Detailed code for data wrangling (“Coral Protection Outcomes_Wrangle.Rmd”) as well as analysis and figure generation (“Coral Protection Outcomes_FinguresAnalysis.Rmd”). Outputs from the data wrangling step to be used in the analysis script are included in the “CoralProtection.Rdata” file.
(ZIP)

S1 Fig COTS strategic management conceptual framework. Diagrammatic representation of COTS outbreak and coral cover dynamics, and the management objectives at various stages of the outbreak cycle. The COTS Control Program capacity and the timing of culling commencement strongly influences when and how the objectives can be achieved.
(TIF)

S2 Fig Spatial scales overview. The spatial relationship between the Great Barrier Reef Marine Park (Marine Park), Sectors, Reefs, and Sites. (A) the entire Marine Park, (B) an individual Sector (Townsville), (C) an individual reef (John Brewer Reef), with an indicative reef-wide manta-tow path as conducted by the AIMS LTMP (D) culling sites.
(TIF)

S3 Fig Sector-wide outbreak definitions and non-outbreak populations. The purple line represents the COTS density at a given location. (A)–An example of a population that would be categorised as an Outbreak. This is due to the population breaching the 0.22 COTS/tow density (an established outbreak). The years categorised as an ‘outbreak’ are denoted by the transparent, red rectangle. The year prior to the outbreak threshold being breached is included to capture the pre-outbreak coral cover and the outbreak is ended with two consecutive years below the threshold. (B)–an example of a population that would not be categorised as an Outbreak. This is due to the density of COTS not crossing the 0.22 COTS/tow threshold (denoting a ‘severe’ outbreak). NB These sector-wide definitions of outbreak periods are distinct from the reef level outbreak which simply reflect the Outbreak Status of a reef at a singular point in time.
(TIF)

S4 Fig Relative coral cover change time series with outlier reefs. Relative change in coral cover (%) by Sector, coloured according to the type of management action implemented ‘Limited Action’ (orange), ‘Reactive’ (yellow), and ‘Timely’ (green) Each line represents the relative change in coral cover at a given reef, up to 6 years following the start of the sector-specific 4^th^ outbreak (see Table 1). Highlighted reefs are example outliers that are discussed in section 3.2. These individual trajectories underpin the modelled trajectory in Fig 4. NB Reefs surveyed less than 3 times were not included in this figure to increase the clarity of individual trajectories. Additionally, each facet is displayed on a variable y-axis to increase visual interpretation. Reefs from “Proactive action” (Cape Upstart sector) are not included in either time series analyses (see Fig 4) as the time series is not long enough from the predicted onset of the outbreak in 2020.
(TIF)

S5 Fig Disturbance regimes of the GBR by sector. (A) The percentage of reefs affected by the 3 major disturbances (COTS, Cyclones and Bleaching) from 1990 as observed by the AIMS LTMP broadscale manta tow surveys. Light and dark grey bars indicate the 3^rd^ and 4^th^ COTS outbreak waves respectively (B). The average coral loss per survey observed within the 3^rd^ and 4^th^ COTS outbreak waves for each of the three major disturbances.
(TIF)

S1 File (DOCX)

## References

[1] Dietzel A, Connolly SR, Hughes TP, Bode M. The spatial footprint and patchiness of large‐scale disturbances on coral reefs. Glob Change Biol. 2021; 27(19):4825–4838. doi: 10.1111/gcb.15805 34390297

[2] Brown BE. Disturbances to reefs in recent times. Life and death of coral reefs 1997:354–379.

[3] Graham NAJ, Chong-Seng KM, Huchery C, Januchowski-Hartley FA, Nash KL. Coral reef community composition in the context of disturbance history on the Great Barrier Reef, Australia. PLoS ONE 2014;9(7):e101204. doi: 10.1371/journal.pone.0101204 24983747 PMC4077760

[4] Gouezo M, Golbuu Y, Fabricius K, Olsudong D, Mereb G, Nestor V, et al. Drivers of recovery and reassembly of coral reef communities. Proceedings of the Royal Society B 2019;286(1897):20182908. doi: 10.1098/rspb.2018.2908 30963834 PMC6408889

[5] McWilliam M, Pratchett MS, Hoogenboom MO, Hughes TP. Deficits in functional trait diversity following recovery on coral reefs. Proceedings of the Royal Society B 2020;287(1918):20192628. doi: 10.1098/rspb.2019.2628 31910784 PMC7003456

[6] Hughes TP, Barnes ML, Bellwood DR, Cinner JE, Cumming GS, Jackson JBC, et al. Coral reefs in the Anthropocene. Nature 2017;546:82–90. doi: 10.1038/nature22901 28569801

[7] Pratchett MS, Cumming GS. Managing cross-scale dynamics in marine conservation: pest irruptions and lessons from culling of crown-of-thorns starfish (*Acanthaster spp*.). Biol Conserv 2019;238:108211.

[8] Anthony KRN, Marshall PA, Abdullah A, Beeden R, Bergh C, Black R, et al. Operationalizing resilience for adaptive coral reef management under global environmental change. Global Change Biol 2015;21(1):48–61. doi: 10.1111/gcb.12700 25196132 PMC4310291

[9] McLeod E, Anthony K, Mumby P, Maynard J, Beeden R, Graham N, et al. The future of resilience-based management in coral reef ecosystems. J Environ Manage 2019;233:291–301. doi: 10.1016/j.jenvman.2018.11.034 30583103

[10] Hughes TP, Graham NAJ, Jackson JBC, Mumby PJ, Steneck RS. Rising to the challenge of sustaining coral reef resilience. Trends Ecol Evol 2010;25(11):633–642. doi: 10.1016/j.tree.2010.07.011 20800316

[11] Osborne K, Dolman AM, Burgess SC, Johns KA. Disturbance and the dynamics of coral cover on the Great Barrier Reef (1995–2009). PloS one 2011;6(3):e17516. doi: 10.1371/journal.pone.0017516 21423742 PMC3053361

[12] Sweatman H, Delean S, Syms C. Assessing loss of coral cover on Australia’s Great Barrier Reef over two decades, with implications for longer-term trends. Coral Reefs 2011;30(2):521–531.

[13] De’ath G, Fabricius KE, Sweatman H, Puotinen M. The 27-year decline of coral cover on the Great Barrier Reef and its causes. Proc Natl Acad Sci USA 2012;109(44):17995–17999. doi: 10.1073/pnas.1208909109 23027961 PMC3497744

[14] Pratchett MS, Caballes CF, Rivera-Posada JA, Sweatman HPA. Limits to understanding and managing outbreaks of crown-of-thorns starfish (*Acanthaster spp*.). Oceanogr Mar Biol Annu Rev 2014;52:133–200.

[15] Endean R. Report on Investigations Made into Aspects of the Current *Acanthaster planci* (Crown-of-thorns) Infestations of Certain Reefs of the Great Barrier Reef. Queensland Department of Primary Industries (Fisheries Branch); Brisbane; 1969.

[16] Pratchett MS, Caballes CF, Cvitanovic C, Raymundo ML, Babcock RC, Bonin MC, et al. Knowledge gaps in the biology, ecology, and management of the Pacific crown-of-thorns sea star *Acanthaster sp*. on Australia’s Great Barrier Reef. Biol Bull 2021;241(3):330–346. 35015620 10.1086/717026

[17] Pratchett MS, Nadler LE, Burn D, Lang BJ, Messmer V, Caballes CF. Reproductive investment and fecundity of Pacific crown-of-thorns starfish (*Acanthaster* cf. *solaris*) on the Great Barrier Reef. Mar Biol 2021;168(6):1–8.

[18] Kayal M, Vercelloni J, Lison de Loma T, Bosserelle P, Chancerelle Y, Geoffroy S, et al. Predator Crown-of-Thorns starfish (*Acanthaster planci*) outbreak, mass mortality of corals, and cascading effects on reef fish and benthic communities. PLoS ONE 2012;7(10). 10.1371/journal.pone.0047363 PMC3466260 23056635

[19] Wooldridge SA, Brodie JE. Environmental triggers for primary outbreaks of crown-of-thorns starfish on the Great Barrier Reef, Australia. Mar Pollut Bull 2015;101(2):805–814. doi: 10.1016/j.marpolbul.2015.08.049 26460182

[20] Brinkman R, Wolanski E, Deleersnijder E, McAllister F, Skirving W. Oceanic inflow from the Coral Sea into the Great Barrier Reef. Estuar Coast Shelf Sci 2002;54(4):655–668.

[21] Vanhatalo J, Hosack GR, Sweatman H. Spatiotemporal modelling of crown‐of‐thorns starfish outbreaks on the Great Barrier Reef to inform control strategies. J Appl Ecol 2017;54(1):188–197.

[22] Engelhardt U, Lassig B. GBRMPA Workshop Series; The Possible Causes and Consequences of Outbreaks of the Crown-of-thorns Starfish: Proceedings of a Workshop held in Townsville, Queensland, Australia, Great Barrier Reef Marine Park Authority 1993.

[23] Authority GBRMP. Crown-of-thorns starfish Strategic Management Framework. Great Barrier Reef Marine Park Authority; 2020.

[24] Birkeland C, Lucas J. *Acanthaster planci*: major management problem of coral reefs. CRC press; 1990.

[25] Association of Marine Park Tourism Operators. COTS Control Program on the Great Barrier Reef: Final Performance Report 2002–2007. Association of Marine Park Tourism Operators Ltd 2007:24pp.

[26] Rivera-Posada J, Pratchett MS, Aguilar C, Grand A, Caballes CF. Bile salts and the single-shot lethal injection method for killing crown-of-thorns sea stars *(Acanthaster planci)*. Ocean Coast Manage 2014;102(Part A):383–390.

[27] Boström-Einarsson L, Rivera-Posada J. Controlling outbreaks of the coral-eating crown-of-thorns starfish using a single injection of common household vinegar. Coral Reefs 2016;35(1):223–228.

[28] Hock K, Wolff NH, Condie SA, Anthony KRN, Mumby PJ. Connectivity networks reveal the risks of crown-of-thorns starfish outbreaks on the Great Barrier Reef. J Appl Ecol 2014;51(5):1188–1196.

[29] Hock K, Wolff NH, Ortiz JC, Condie SA, Anthony KRN, Blackwell PG, et al. Connectivity and systemic resilience of the Great Barrier Reef. PLoS Biology 2017;15(11):e2003355. doi: 10.1371/journal.pbio.2003355 29182630 PMC5705071

[30] Fletcher CS, Westcott DA, Bonin MC. An ecologically-based operational strategy for COTS control: integrated decision making from the site to the regional scale. Report to the National Environmental Science Programme. Reef and Rainforest Research Centre Limited, Cairns. 2020.

[31] Westcott DA, Fletcher CS, Babcock R, Pláganyi Lloyd É. A strategy to link research and management of crown-of-thorns starfish on the Great Barrier Reef: an integrated pest management approach. Report to the National Environmental Science Programme. Reef and Rainforest Research Centre Limited, Cairns 2016:1–80.

[32] Westcott DA, Fletcher CS, Gladish DW, MacDonald S, Condie S. Integrated pest management crown-of-thorns starfish control program on the Great Barrier Reef: current performance and future potential. Report to the National Environmental Science Program. Reef and Rainforest Research Centre Limited, Cairns 2021.

[33] Commonwealth of Australia. Reef 2050 Long-Term Sustainability Plan 2021–2025. 2021.

[34] Great Barrier Reef Marine Park Authority. Great Barrier Reef blueprint for resilience. 2017.

[35] Plagányi ÉE, Babcock RC, Rogers J, Bonin M, Morello E. Ecological analyses to inform management targets for the culling of crown-of-thorns starfish to prevent coral decline. Coral Reefs 2020;39(5):1483–1499.

[36] Rogers J, Pláganyi É, Babcock R. Aggregation, Allee effects and critical thresholds for the management of the crown-of-thorns starfish *Acanthaster planci*. Marine ecology. Progress series (Halstenbek) 2017;578:99–114.

[37] Westcott DA, Fletcher CS, Kroon FJ, Babcock RC, Plagányi EE, Pratchett MS, et al. Relative efficacy of three approaches to mitigate crown-of-thorns starfish outbreaks on Australia’s Great Barrier Reef. Scientific reports 2020;10(1):1–12. 32724152 10.1038/s41598-020-69466-1 PMC7387460

[38] Emslie MJ, Bray P, Cheal AJ, Johns KA, Osborne K, Sinclair-Taylor T, et al. Decades of monitoring have informed the stewardship and ecological understanding of Australia’s Great Barrier Reef. Biological conservation 2020;252:108854.

[39] Spalding M, Burke L, Wood SA, Ashpole J, Hutchison J, Zu Ermgassen P. Mapping the global value and distribution of coral reef tourism. Mar Policy 2017;82:104–113.

[40] Miller IR, Jonker M, Coleman G. Crown-of-thorns starfish and coral surveys using the manta tow technique. Long-term monitoring of the Great Barrier Reef standard operational procedure. 2019;9:1–41.

[41] De’ath G, CRC Reef Research Centre. Analyses of Crown-of-thorns Starfish Data from the Fine-scale Surveys and Long-term Monitoring Program Manta Tow Surveys. CRC Reef Research Centre; 2003.

[42] R Core Team. R: a language and environment for statistical computing. 2013.

[43] Wickham H, Averick M, Bryan J, Chang W, McGowan LD, François R, et al. Welcome to the Tidyverse. Journal of open source software 2019;4(43):1686.

[44] Bürkner P. brms: An R package for Bayesian multilevel models using Stan. Journal of statistical software 2017;80:1–28.

[45] Hartig F, Hartig MF. Package ‘DHARMa’. R package 2017.

[46] Russell V. L. emmeans: Estimated Marginal Means, aka Least-Squares Means. R package version 1.8.5. 2023.

[47] Wickham H. ggplot2: elegant graphics for data analysis 2016.

[48] Rue H, Martino S, Chopin N. Approximate Bayesian inference for latent Gaussian models by using integrated nested Laplace approximations. Journal of the Royal Statistical Society Series B: Statistical Methodology 2009;71(2):319–392.

[49] Ceccarelli DM, Emslie MJ, Richards ZT. Post-disturbance stability of fish assemblages measured at coarse taxonomic resolution masks change at finer scales. PLoS One 2016;11(6):e0156232. doi: 10.1371/journal.pone.0156232 27285160 PMC4902313

[50] Hughes TP, Kerry JT, Ãlvarez-Noriega M, Ãlvarez-Romero JG, Anderson KD, Baird AH, et al. Global warming and recurrent mass bleaching of corals. Nature 2017;543:373–377. doi: 10.1038/nature21707 28300113

[51] Castro‐Sanguino C, Bozec Y, Condie SA, Fletcher CS, Hock K, Roelfsema C, et al. Control efforts of crown‐of‐thorns starfish outbreaks to limit future coral decline across the Great Barrier Reef. Ecosphere 2023;14(6):e4580.

[52] Condie SA, Anthony KRN, Babcock RC, Baird ME, Beeden R, Fletcher CS, et al. Large-scale interventions may delay decline of the Great Barrier Reef. Royal Society Open Science 2021;8(4):201296. doi: 10.1098/rsos.201296 34007456 PMC8080001

[53] Fletcher CS, Castro-Sanguino C, Condie S, Bozec Y, Hock K, Gladish DW, et al. Regional-scale modelling capability for assessing crown-of-thorns starfish control strategies on the Great Barrier Reef. Report to the National Environmental Science Program. Reef and Rainforest Research Centre Limited 2021:59pp.

[54] Babcock RC, Plagányi ÉE, Condie SA, Westcott DA, Fletcher CS, Bonin MC, et al. Suppressing the next crown-of-thorns outbreak on the Great Barrier Reef. Coral Reefs 2020;39(5):1233–1244.

[55] Rogers JG, Plagányi ÉE. Culling corallivores improves short-term coral recovery under bleaching scenarios. Nature communications 2022;13(1):1–17. 10.1038/s41467-022-30213-x PMC9085818 35534497

[56] Hughes TP, Kerry JT, Simpson T. Large-scale bleaching of corals on the Great Barrier Reef. Ecology 2018;99(2):501. doi: 10.1002/ecy.2092 29155453

[57] Hughes TP, Baird AH, Dinsdale EA, Motschaniwskyj NA, Pratchett MS, Tanner JE, et al. Supply-side ecology works both ways: The link between benthic adults, fecundity, and larval recruits. Ecology 2000 Aug;81(8):2241–2249.

[58] Australian Institute of Marine Science. Long-Term Monitoring Program Annual Summary Report of Coral Reef Condition 2021/22. Australian Institute of Marine Science 2022: 1–11.

[59] Babcock RC, Milton DA, Pratchett MS. Relationships between size and reproductive output in the crown-of-thorns starfish. Mar Biol 2016;163(11):234.

[60] Desbiens A, Roff G, Razak TB, Wolfe K, Emslie MJ, Hoey A, et al. Integrative carbonate budget model of the Great Barrier Reef. Reef and Rainforest Research Centre Limited, Cairns 2021.

[61] Kennedy EV, Perry CT, Halloran PR, Iglesias-Prieto R, Schönberg CHL, Wisshak M, et al. Avoiding coral reef functional collapse requires local and global action. Current Biology 2013;23(10):912–918. doi: 10.1016/j.cub.2013.04.020 23664976

[62] Perry CT, Murphy GN, Kench PS, Smithers SG, Edinger EN, Steneck RS, et al. Caribbean-wide decline in carbonate production threatens coral reef growth. Nature Communications 2013;4:1402. doi: 10.1038/ncomms2409 23360993 PMC3660652

[63] Ortiz J, Wolff NH, Anthony KR, Devlin M, Lewis S, Mumby PJ. Impaired recovery of the Great Barrier Reef under cumulative stress. Science advances 2018;4(7):6127. doi: 10.1126/sciadv.aar6127 30035217 PMC6051737

[64] Pratchett MS. Changes in coral assemblages during an outbreak of *Acanthaster planci* at Lizard Island, northern Great Barrier Reef (1995–1999). Coral Reefs 2010;29(3):717–725.

[65] Kleypas J, Allemand D, Anthony K, Baker AC, Beck MW, Hale LZ, et al. Designing a blueprint for coral reef survival. Biol Conserv 2021;257:109107.

[66] van Hooidonk R, Maynard J, Tamelander J, Gove J, Ahmadia G, Raymundo L, et al. Local-scale projections of coral reef futures and implications of the Paris agreement. Scientific Reports 2016;6:1–8. 28000782 10.1038/srep39666 PMC5175274

[67] Baker AC, Glynn PW, Riegl B. Climate change and coral reef bleaching: an ecological assessment of long-term impacts, recovery trends and future outlook. Estuar Coast Shelf Sci 2008;80(4):435–471.

[68] Dixon AM, Puotinen M, Ramsay HA, Beger M. Coral reef exposure to damaging tropical cyclone waves in a warming climate. Earth’s Future 2022;10(8):e2021EF002600.

[69] Knutson TR, McBride JL, Chan J, Emanuel K, Holland G, Landsea C, et al. Tropical cyclones and climate change. Nature Geoscience 2010;3:157–163.

[70] Randall CJ, Negri AP, Quigley KM, Foster T, Ricardo GF, Webster NS, et al. Sexual production of corals for reef restoration in the Anthropocene. Mar Ecol Prog Ser 2020;635:203–232.

[71] van Oppen MJH, Gates RD, Blackall LL, Cantin N, Chakravarti LJ, Chan WY, et al. Shifting paradigms in restoration of the world’s coral reefs. Global change biology 2017 Sep;23(9):3437–3448. doi: 10.1111/gcb.13647 28247459

[72] van Oppen MJH, Oliver JK, Putnam HM, Gates RD. Building coral reef resilience through assisted evolution. Proceedings of the National Academy of Sciences—PNAS 2015;112(8):2307–2313. doi: 10.1073/pnas.1422301112 25646461 PMC4345611

[73] Bayraktarov E, Saunders MI, Abdullah S, Mills M, Beher J, Possingham HP, et al. The cost and feasibility of marine coastal restoration. Ecol Appl 2016;26(4):1055–1074. doi: 10.1890/15-1077 27509748

[74] Haisfield KM, Fox HE, Yen S, Mangubhai S, Mous PJ. An ounce of prevention: cost‐effectiveness of coral reef rehabilitation relative to enforcement. Conservation Letters 2010;3(4):243–250.

[75] Hughes TP, Kerry JT, Connolly SR, Baird AH, Eakin CM, Heron SF, et al. Ecological memory modifies the cumulative impact of recurrent climate extremes. Nature Climate Change 2019;9:40–43.

[76] Marzonie MR, Bay LK, Bourne DG, Hoey AS, Matthews S, Nielsen JJ, et al. The effects of marine heatwaves on acute heat tolerance in corals. Global Change Biol 2023;29(2):404–416. doi: 10.1111/gcb.16473 36285622 PMC10092175

[77] Mellin C, Matthews S, Anthony KRN, Brown SC, Caley MJ, Johns K, et al. Spatial resilience of the Great Barrier Reef under cumulative disturbance impacts. Global Change Biol 2019;25:2431–2445. doi: 10.1111/gcb.14625 30900790

[78] Mendelsohn R, Emanuel K, Chonabayashi S, Bakkensen L. The impact of climate change on global tropical cyclone damage. Nature climate change 2012;2(3):205–209.

[79] McWhorter JK, Halloran PR, Roff G, Skirving WJ, Perry CT, Mumby PJ. The importance of 1.5°C warming for the Great Barrier Reef. Global Change Biol 2022;28(4):1332–1341. 10.1111/gcb.15994 34783126

[80] Australian Institue of Marine Science. Long-term Reef Monitoring Program: Annual Summary Report on Coral Reef Condition for 2022/23. 2023:11.

[81] Wilmes JC, Caballes CF, Cowan Z, Hoey AS, Lang BJ, Messmer V, et al. Contributions of pre-versus post-settlement processes to fluctuating abundance of crown-of-thorns starfishes (*Acanthaster spp*.). Mar Pollut Bull 2018;135:332–345. 30301045 10.1016/j.marpolbul.2018.07.006

[82] Lang BJ, Donelson JM, Caballes CF, Uthicke S, Doll PC, Pratchett MS. Effects of elevated temperature on the performance and survival of pacific crown-of-thorns starfish (*Acanthaster cf*. *solaris*). Mar Biol 2022;169(4):1–13.

[83] Matthews SA, Mellin C, Pratchett MS. Larval connectivity and water quality explain spatial distribution of crown-of-thorns starfish outbreaks across the Great Barrier Reef. Advances in Marine Biology: Elsevier; 2020. p. 223–258. 10.1016/bs.amb.2020.08.007 33293012

[84] Wilmes JC, Schultz DJ, Hoey AS, Messmer V, Pratchett MS. Habitat associations of settlement-stage crown-of-thorns starfish on Australia’s Great Barrier Reef. Coral Reefs 39 (2020): 1163–1174; doi: 10.1007/s00338-6

[85] Wilmes JC, Hoey AS, Pratchett MS. Contrasting size and fate of juvenile crown-of-thorns starfish linked to ontogenetic diet shifts. Proceedings of the Royal Society B 2020;287(1931):20201052. doi: 10.1098/rspb.2020.1052 32693724 PMC7423671

[86] Pratchett MS, Caballes CF, Burn D, Doll PC, Chandler JF, Doyle JR, et al. Scooter-assisted large area diver-based (SALAD) visual surveys to test for renewed outbreaks of crown-of-thorns starfish (*Acanthaster* cf. *solaris*) in the northern Great Barrier Reef. A report to the Australian Government by the COTS Control Innovation Program 2022:32.

[87] Miller I, Sweatman H, Cheal A, Emslie M, Johns K, Jonker M, et al. Origins and implications of a primary crown-of-thorns starfish outbreak in the Southern Great Barrier Reef. Journal of Marine Biology 2015;2015:809624.

[88] Reichelt RE, Bradbury RH, Moran PJ. Distribution of *Acanthaster planci* outbreaks on the Great Barrier Reef between 1966 and 1989. Coral Reefs 1990;9:97–103.

[89] Kroon FJ, Barneche DR, Emslie MJ. Fish predators control outbreaks of Crown-of-Thorns Starfish. Nature Communications 2021;12(1):6986. doi: 10.1038/s41467-021-26786-8 34880205 PMC8654818

[90] Fabricius KE, Okaji K, De’Ath G. Three lines of evidence to link outbreaks of the crown-of-thorns seastar *Acanthaster planci* to the release of larval food limitation. Coral Reefs 2010;29(3):593–605.

[91] Birkeland C. Terrestrial runoff as a cause of outbreaks of *Acanthaster planci* (Echinodermata: Asteroidea). Mar Biol 1982;69:175–185.

[92] Brodie J, Devlin M, Lewis S. Potential enhanced survivorship of crown of thorns starfish larvae due to near-annual nutrient enrichment during secondary outbreaks on the central mid-shelf of the Great Barrier Reef, Australia. Diversity 2017;9(1):17.

[93] Uthicke S, Lamare M, Doyle JR. eDNA detection of corallivorous seastar (*Acanthaster cf*. *solaris*) outbreaks on the Great Barrier Reef using digital droplet PCR. Coral Reefs 2018;37(4):1229–1239.

[94] Uthicke S, Robson B, Doyle JR, Logan M, Pratchett MS, Lamare M. Developing an effective marine eDNA monitoring: eDNA detection at pre-outbreak densities of corallivorous seastar (*Acanthaster* cf. *solaris*). Sci Total Environ 2022;851:158143. 35995149 10.1016/j.scitotenv.2022.158143

[95] Hall MR, Kocot KM, Baughman KW, Fernandez-Valverde SL, Gauthier ME, Hatleberg WL, et al. The crown-of-thorns starfish genome as a guide for biocontrol of this coral reef pest. Nature 2017;544(7649):231–234. doi: 10.1038/nature22033 28379940

