# Supplementary material for: Protecting Great Barrier Reef resilience through effective management of crown-of-thorns starfish outbreaks
Source: PLoS One. 2024 Apr 24;19(4):e0298073. doi: 10.1371/journal.pone.0298073 (PMC11042723; doi:10.1371/journal.pone.0298073)
Supplement: S1 Appendix — Detailed explanations of the use of disturbance data, COTS control program methods, definition of sector-wide management action and the rationale for categorising the Cape Upstart sector as ‘Proactive action’. Also includes supplemental tables: Table S1. Glossary of key words and acronyms; Table S2. Model formulae and the description of the variables analysed; Table S3. Summary statistics for COTS densities and Coral Cover for each outbreak by sector. (DOCX) [file pone.0298073.s001.docx]

# Supporting Information

## S1 Appendix - Supplementary Text

### Disturbance data

During AIMS LTMP manta tow observations, causes of mortality are attributed to significant coral losses (>5%) where possible. For Fig S5 (A) the percentage of reefs affected by each major disturbance (COTS, Bleaching, Storms) by sector was calculated each year using this attribution. For clarity, unknown disturbances were excluded as were coral disease due to the low prevalence in the dataset. For Fig S5 (B) the coral loss per survey was calculated as the mean +/- standard error of coral loss recorded from the year prior to the mortality event being observed for each disturbance type by sector. This metric includes all reefs that were surveyed and not just those affected by disturbance to provide a clear estimate of the prevalence and magnitude of coral losses from each disturbance.

### COTS control methods

COTS culling occurs in a highly organized and structured manner to ensure the most effective use of resources. Currently, multiple contractors (some with multiple vessels) perform culling following the Integrated Pest Management framework. This framework allows managers to make informed decisions about where to prioritise culling effort and when culling can cease at certain reefs.

Briefly put, each targeted reef is allocated to a specific contractor who exclusively perform the culling at that reef. First, the reef is surveyed using a manta tow during which a diver is pulled behind a small tender around the reef perimeter. Tows are broken into approximately 200m segments during which a diver records: 1) the estimated coral cover, 2) the number of COTS feeding scars, and 3) the number of COTS observed. This data is then paired with the GPS track recorded for each tow and is used to inform where culling should be performed.

Every actioned reef is broken into smaller management units called “sites” which are approximately 500m long and 200m wide. All culling is performed and recorded at the site level. Using a mix of snorkeling and SCUBA, a team of divers swim along the site in a line covering the full spread of reef habitats (slope, crest, and flat) and aim to completely survey the site within a fixed amount time (typically 40-60 minutes). Divers search for COTS and inject any located individuals with 10mL of ox bile solution or vinegar. This injection causes rapid disease formation and subsequent death in the following 24-48 hours. The number of COTS and their respective size cohort (<15cm, 15-25cm, 25-40cm, and >40cm) culled at each site is recorded after every dive. The catch per unit effort (CPUE: total number of COTS >15cm culled/total minutes spent culling) is calculated using the culling data for every site actioned. The CPUE is then used to determine if culling needs to be continued at that site (≥0.04, i.e. the site remains open) or if the site is then closed to culling (<0.04) as it now has an ecologically stable population of COTS.

All tow and culling data is uploaded and compiled in a central database after every voyage. This data is then made available to the contractors through an interactive dashboard that is referenced during subsequent voyages. Governing and management authorities also use this data to study trends and make informed decisions as to how to change or maintain the culling program.

### Categorising the Cape Upstart sector as ‘Proactive action’

The 4^th^ outbreak defined for the Cape Upstart sector is a forecast based upon historic outbreak cycles. The years that denote this outbreak are based on the southwardly progression of COTS and the observed 4-year delay between the 3^rd^ outbreak commencing in the Townsville sector (1998) and the Cape Upstart sector (2002). This expected outbreak was denoted only because this sector had demonstrated a history of previous outbreaks, thus allowing us to assume that there would be a current outbreak.

### ****Detailed definition of sector-wide management action****

1. ***Limited action:*** When limited resources are available and/or control activities were not initiated early enough COTS outbreaks may become too severe to control at both the reef and sector wide level. These reefs/sectors are typified by rapid coral loss beyond the resilience threshold with longer recovery times expected.
2. ***Reactive action:*** Control begins either after COTS outbreaks have exceeded severe outbreak levels or before this threshold is reached, but limited resources does not allow for the suppression of the outbreak. Substantially higher amounts of culling resources are required to reduce COTS numbers and prevent coral loss. Coral protection can thus only be achieved at a reduced number of sites and reefs. Coral protection may not be sustainable over the long term as continued COTS larval supply from surrounding reefs may eventually overwhelm control efforts. The goal of protection is to maintain coral cover above the resilience threshold (~10% coral cover, (63) at important source reefs to allow for increased rates of recovery following the outbreak (e.g., increased coral larvae export).
3. ***Timely action:*** Culling begins after reaching Potential outbreak densities >0.11 with sufficient resources to suppress the COTS populations. Suppression greatly reduces the regional spawning potential of COTS populations and a reduction in coral predation. This has positive consequences for both the culled reefs and those with downstream connectivity by reducing COTS larval supply and increasing coral larval supply.
4. ***Proactive action:*** Proactive action works via two mechanisms: pre-emptive culling targeted before COTS reach outbreak densities (0.11 COTS/Tow) and/or via effective suppression and containment in upstream sectors meaning that a sector wide outbreak does not eventuate. Importantly, proactive action takes place during the phase preceding the outbreak – the non-outbreak phase. The definition of proactive action via upstream suppression is reliant upon the sector in question having a history of sector-wide COTS outbreaks (working off the assumption that if culling did not occur, the sector would not be impacted by an outbreak). Successful proactive action at a reef/sector will have the greatest positive impact on coral growth and recovery. This approach will by definition result in lower total COTS culled and CPUE as efforts are targeted before outbreaks begin. It is important to note that proactive action in this context does not refer to COTS populations being eliminated from a sector. Instead, it simply means that a sector wide outbreak, which would have normally eventuated, has been prevented/delayed due to pre-emptive and/or upstream COTS control.

Table S1. Glossary of key words and acronyms used throughout the text with their definitions.

| **Term** | **Definition** |
| --- | --- |
| Threshold | A specific density of COTS that corresponds to altering important reef properties (e.g. 0.22 COTS/Tow) |
| Sector | Latitudinal bands used by AIMS to categorize reefs and track coral cover at broader scales. There are 11 sectors that span the entire length of the GBR which range in size from 7,500 – 55,000 km^2^. |
| Initiation Box | Reefs between Cairns and Lizard Island where primary outbreaks are believed to commence. |
| Primary Outbreak | The first outbreaks observed within the cycle. These arise independently from other outbreaks and occur on reefs within the initiation box (between Lizard Island and Cairns). |
| Secondary Outbreak | Successive outbreaks that result from increased larval supply from other outbreaks. Waves of secondary outbreaks generally move in a southward direction depending on prevailing currents. |
| GBRMPA | Great Barrier Reef Marine Park Authority. The governing body that manages all activities and operations within the marine park. |
| AIMS | Australian Institute of Marine Science. Government funded research institution focusing on tropical marine ecosystems in Australia. |
| LTMP | The AIMS Long-Term Monitoring Program. This is a large-scale assessment of coral cover on the entire GBR spanning four decades. |
| RRRC | The Reef and Rainforest Research Centre. One of the contractors that solicits operators to conduct the culling. |
| GBRF | The Great Barrier Reef Foundation. A partner that manages the funds used to hire contractors. |
| Severe Outbreak | When the average COTS/Tow for a reef exceeds 1 |
| Established Outbreak | When the average COTS/Tow for a reef range between 0.22 to 1 |
| Potential Outbreak | When the average COTS/Tow for a reef range between 0.11 to 0.22 |

Table S2. Model formulae and the description of the variables analysed.

| **model formula** | **Description** |
| --- | --- |
| COTS density ~ Sector*Outbreak + (1\|Reef) | COTS density as a product of the sector (a proxy for management objective) and outbreak (3^rd^ vs 4^th^) with the interaction thereof. The specific reef is included as a random effect. |
| Δ Coral Cover ~ Sector*Outbreak + (1\|Reef) | Relative change in coral cover as a product of the sector (a proxy for management objective) and outbreak (3^rd^ vs 4^th^) with the interaction thereof. The specific reef is included as a random effect. |
| Δ Coral Cover ~ s(Relative Year) | Relative change in coral cover as a smoothed product of the years since outbreak initiation within the sector. A model was fit to each sector individually and to each management objective. |
| Δ Coral Cover ~ Culling Effort + (1\|Sector) | Absolute change in coral cover as a product of the degree of culling effort applied. The sector from which the reef is from is included as a random effect. |

Table S3. Summary statistics for COTS densities and Coral Cover for each outbreak by sector. The 90% highest posterior density (HPD) intervals are given in parentheses (lower 90% : upper 90%)

|  |  | **COTS Densities** | | **Coral Cover** | |
| --- | --- | --- | --- | --- | --- |
|  | Sector | 3^rd^ Outbreak | 4^th^ Outbreak | 3^rd^ Outbreak | 4^th^ Outbreak |
|  | Cooktown-Lizard Island | 0.43 [0.23:0.69] | 0.90 [0.42:1.48] | -11% [-24:+4] | -41% [-58:-25] |
|  | Cairns | 0.20 [0.08:0.34] | 0.14 [0.04:0.32] | -11% [-29:+8] | +4% [-18:+28] |
|  | Innisfail | 0.69 [0.24:1.30] | 0.50 [0.18:1.02] | -33% [-53:-12] | -28% [-49:-7] |
|  | Townsville | 1.50 [0.52:2.68] | 0.21 [0.07:0.41] | -37% [-51:-20] | +44% [+23:+62] |
|  | Cape Upstart | 0.56 [0.09:1.45] | 0.11 [0.01:0.50] | -31% [-60:+1] | +14% [-33:+61] |
|  | Capricorn Bunker | NA | 0.25 [0.03:0.83] | NA | +46% [+4:+81] |
|  | Swain | 1.45 [0.39:3.18] | 1.72 [0.74:3.34] | -50% [-74:-27] | -46% [-68:-27] |
