# Supplementary figures and images for: Protecting Great Barrier Reef resilience through effective management of crown-of-thorns starfish outbreaks
Source: PLoS One. 2024 Apr 24;19(4):e0298073. doi: 10.1371/journal.pone.0298073 (PMC11042723; doi:10.1371/journal.pone.0298073)

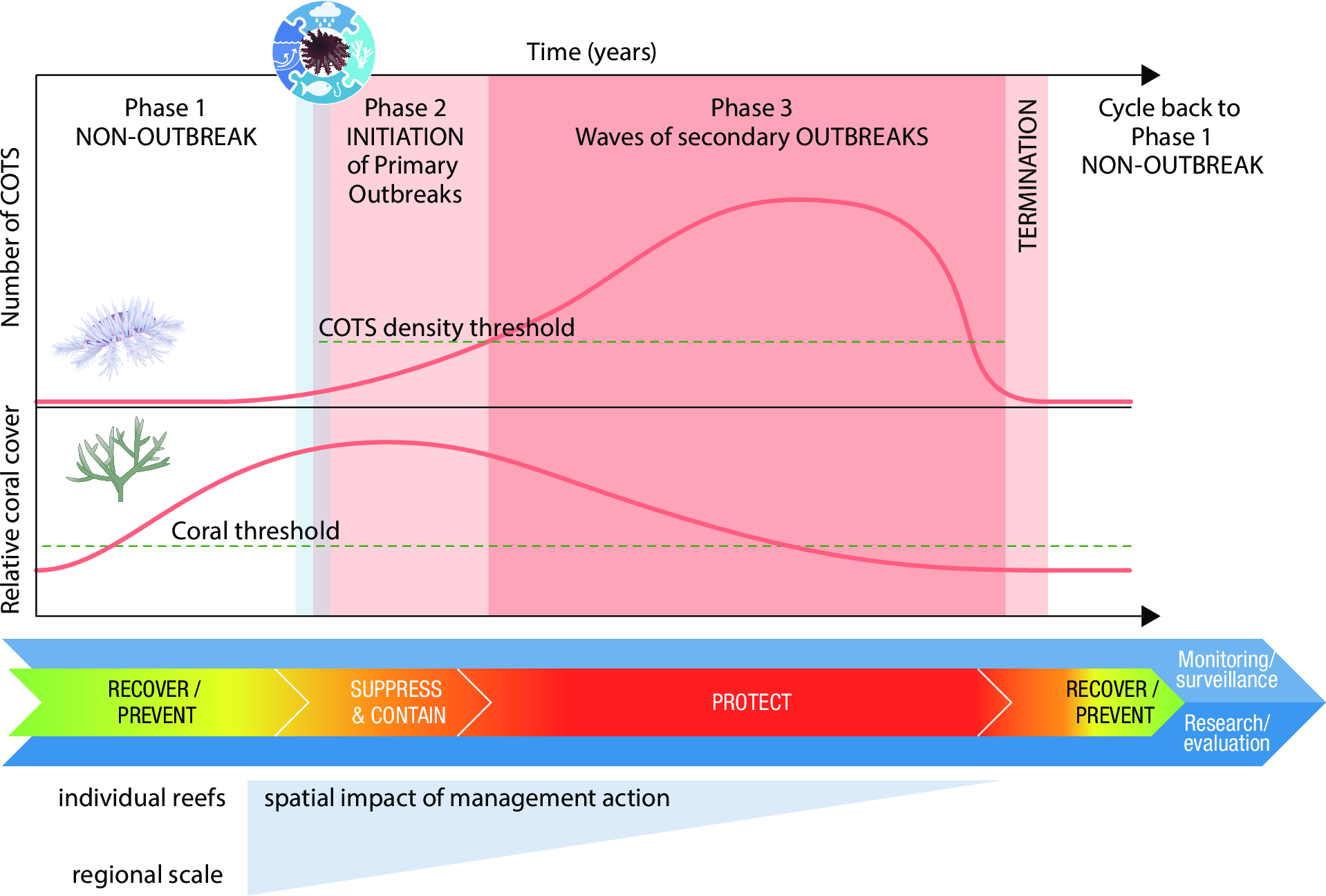

Supplement: S1 Fig — Diagrammatic representation of COTS outbreak and coral cover dynamics, and the management objectives at various stages of the outbreak cycle. The COTS Control Program capacity and the timing of culling commencement strongly influences when and how the objectives can be achieved. (TIF) [file pone.0298073.s003.tif]

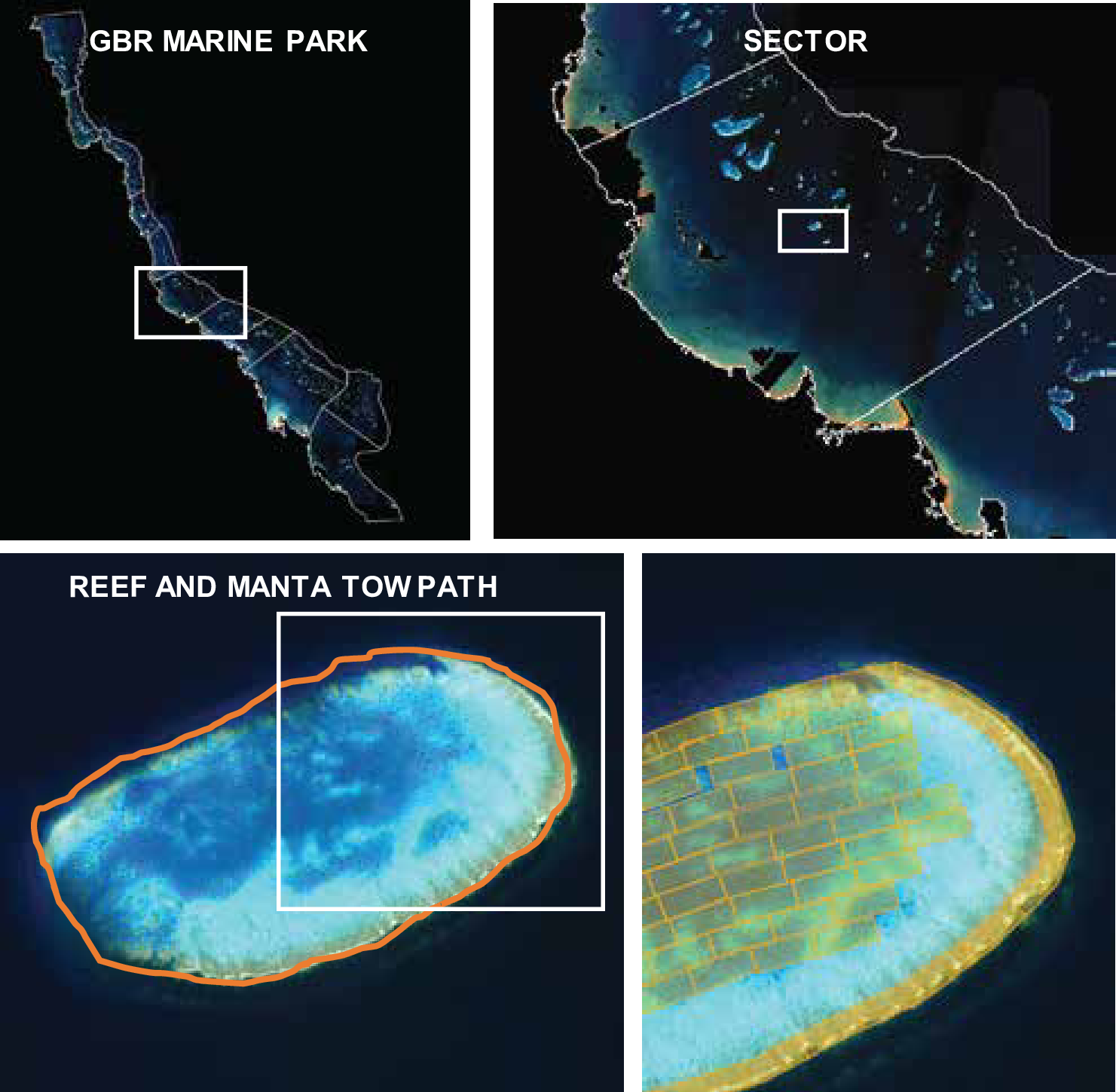

Supplement: S2 Fig — The spatial relationship between the Great Barrier Reef Marine Park (Marine Park), Sectors, Reefs, and Sites. (A) the entire Marine Park, (B) an individual Sector (Townsville), (C) an individual reef (John Brewer Reef), with an indicative reef-wide manta-tow path as conducted by the AIMS LTMP (D) culling sites. (TIF) [file pone.0298073.s004.tif]

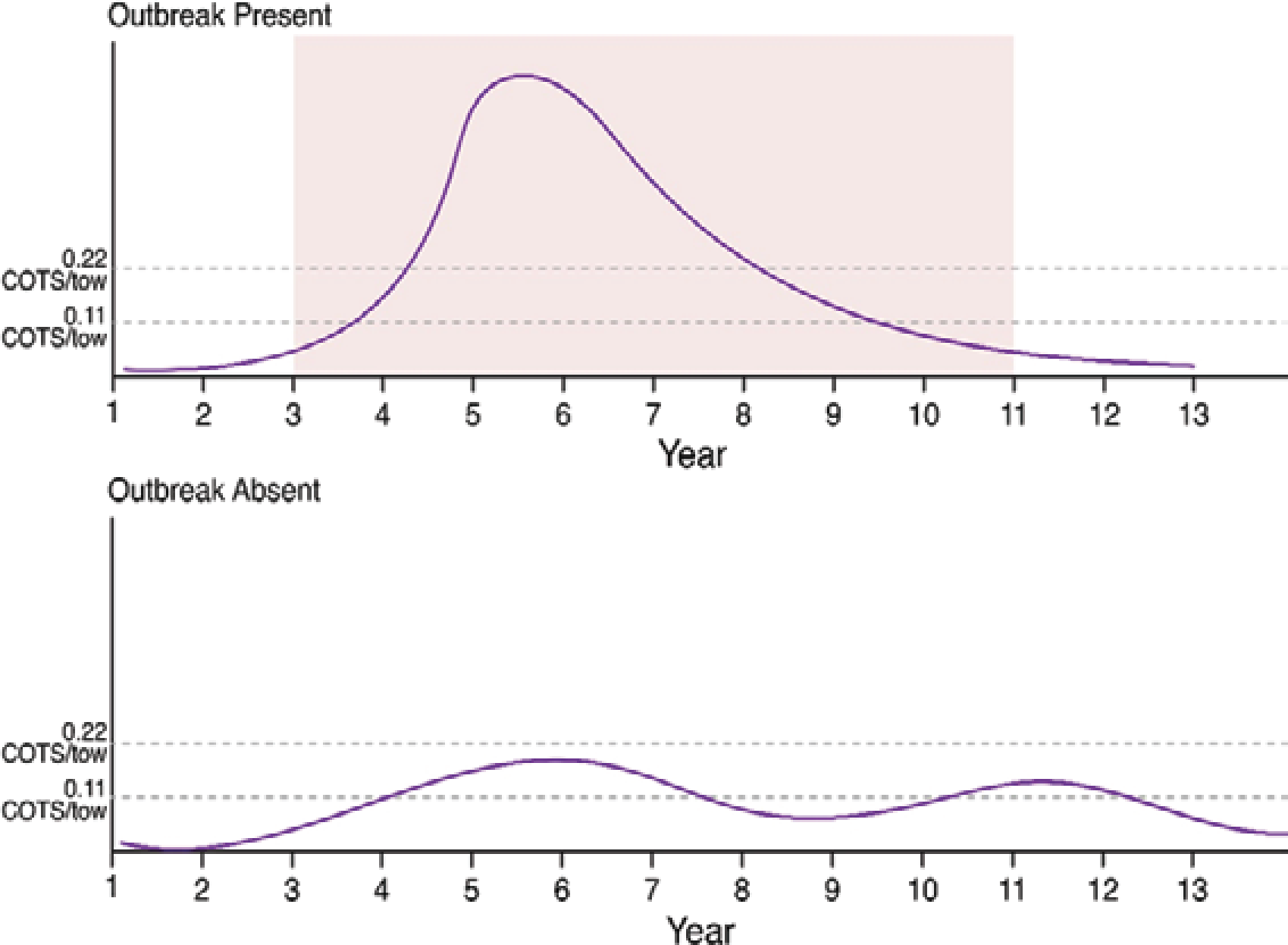

Supplement: S3 Fig — The purple line represents the COTS density at a given location. (A)–An example of a population that would be categorised as an Outbreak. This is due to the population breaching the 0.22 COTS/tow density (an established outbreak). The years categorised as an ‘outbreak’ are denoted by the transparent, red rectangle. The year prior to the outbreak threshold being breached is included to capture the pre-outbreak coral cover and the outbreak is ended with two consecutive years below the threshold. (B)–an example of a population that would not be categorised as an Outbreak. This is due to the density of COTS not crossing the 0.22 COTS/tow threshold (denoting a ‘severe’ outbreak). NB These sector-wide definitions of outbreak periods are distinct from the reef level outbreak which simply reflect the Outbreak Status of a reef at a singular point in time. (TIF) [file pone.0298073.s005.tif]

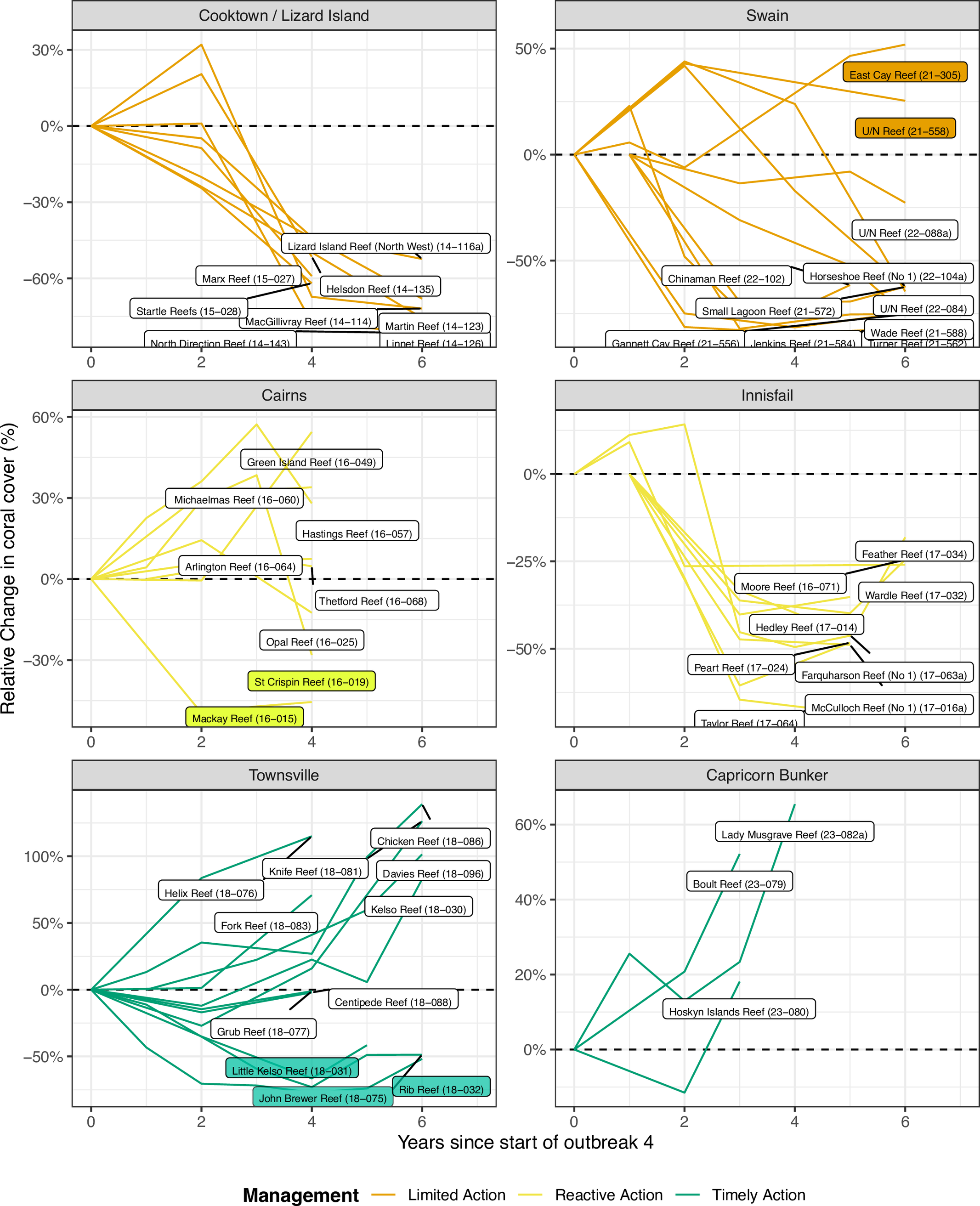

Supplement: S4 Fig — Relative change in coral cover (%) by Sector, coloured according to the type of management action implemented ‘Limited Action’ (orange), ‘Reactive’ (yellow), and ‘Timely’ (green) Each line represents the relative change in coral cover at a given reef, up to 6 years following the start of the sector-specific 4th outbreak (see Table 1). Highlighted reefs are example outliers that are discussed in section 3.2. These individual trajectories underpin the modelled trajectory in Fig 4. NB Reefs surveyed less than 3 times were not included in this figure to increase the clarity of individual trajectories. Additionally, each facet is displayed on a variable y-axis to increase visual interpretation. Reefs from “Proactive action” (Cape Upstart sector) are not included in either time series analyses (see Fig 4) as the time series is not long enough from the predicted onset of the outbreak in 2020. (TIF) [file pone.0298073.s006.tif]

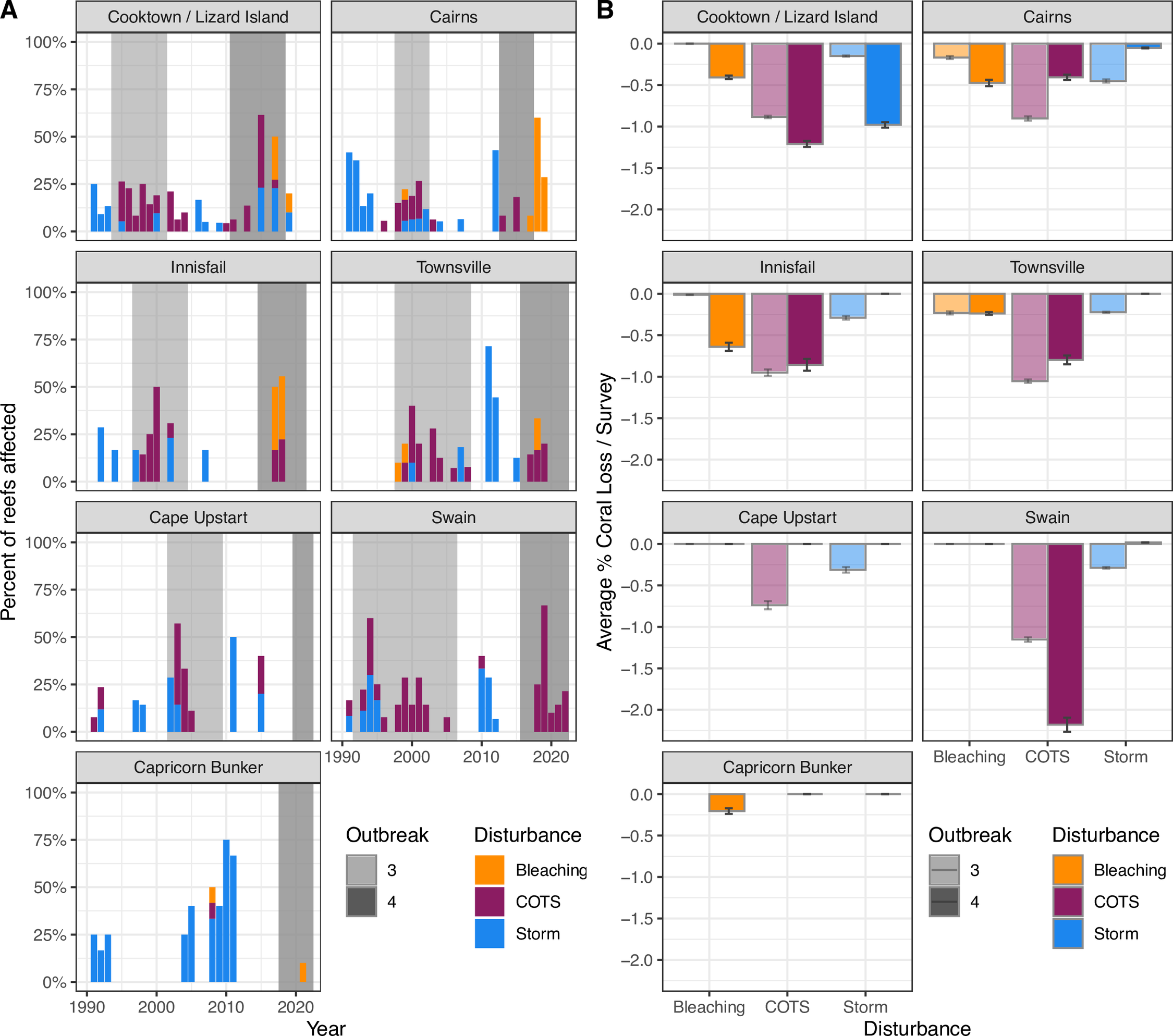

Supplement: S5 Fig — (A) The percentage of reefs affected by the 3 major disturbances (COTS, Cyclones and Bleaching) from 1990 as observed by the AIMS LTMP broadscale manta tow surveys. Light and dark grey bars indicate the 3rd and 4th COTS outbreak waves respectively (B). The average coral loss per survey observed within the 3rd and 4th COTS outbreak waves for each of the three major disturbances. (TIF) [file pone.0298073.s007.tif]
