# Supplementary material for: Protecting Great Barrier Reef resilience through effective management of crown-of-thorns starfish outbreaks
Source: PLoS One. 2024 Apr 24;19(4):e0298073. doi: 10.1371/journal.pone.0298073 (PMC11042723; doi:10.1371/journal.pone.0298073)
Supplement: S1 File — (DOCX) [file pone.0298073.s008.docx]

# Supporting Information

## Supplementary Figures


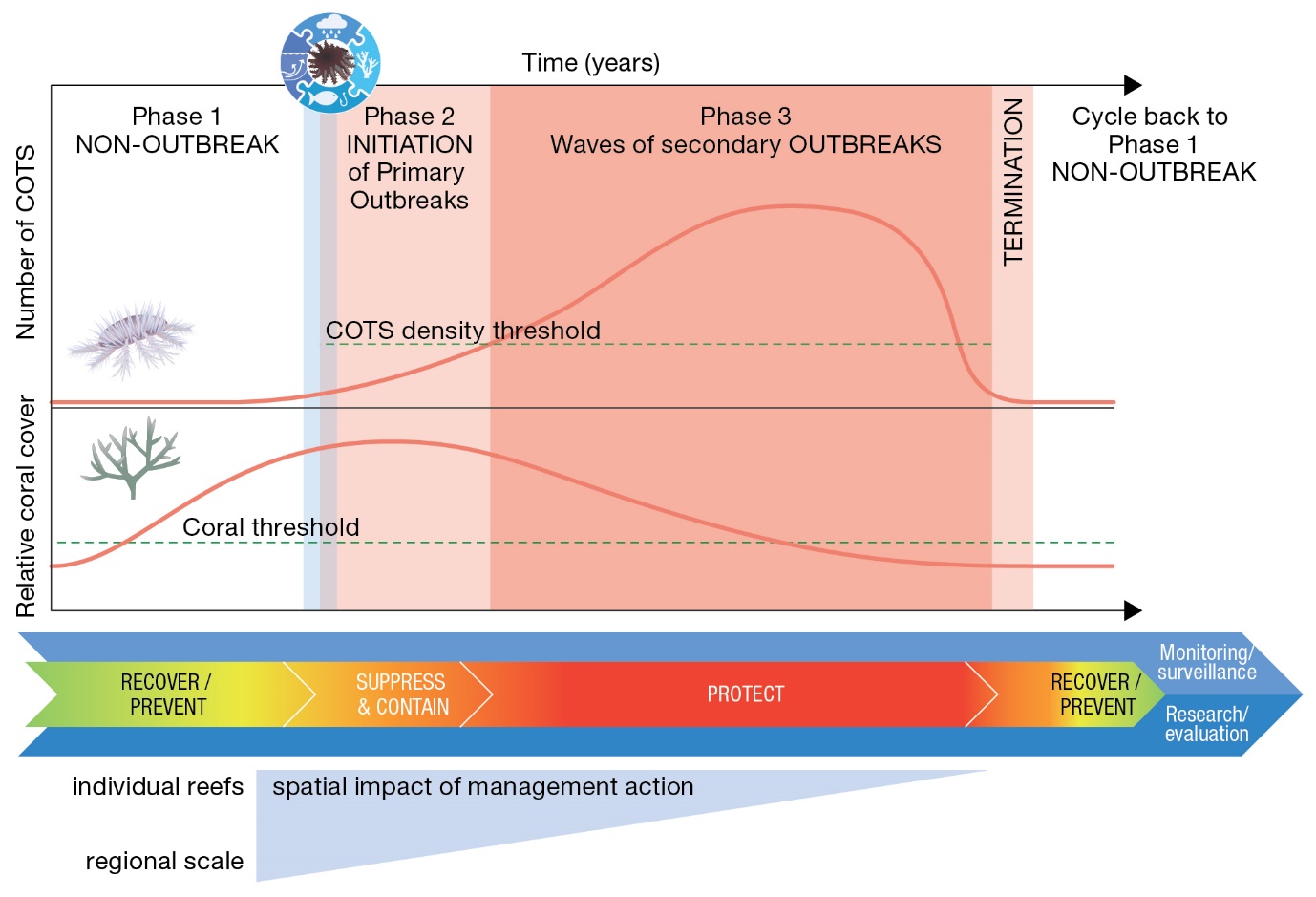


Fig S1. Diagrammatic representation of COTS outbreak and coral cover dynamics, and the management objectives at various stages of the outbreak cycle. The COTS Control Program capacity and the timing of culling commencement strongly influences when and how the objectives can be achieved.


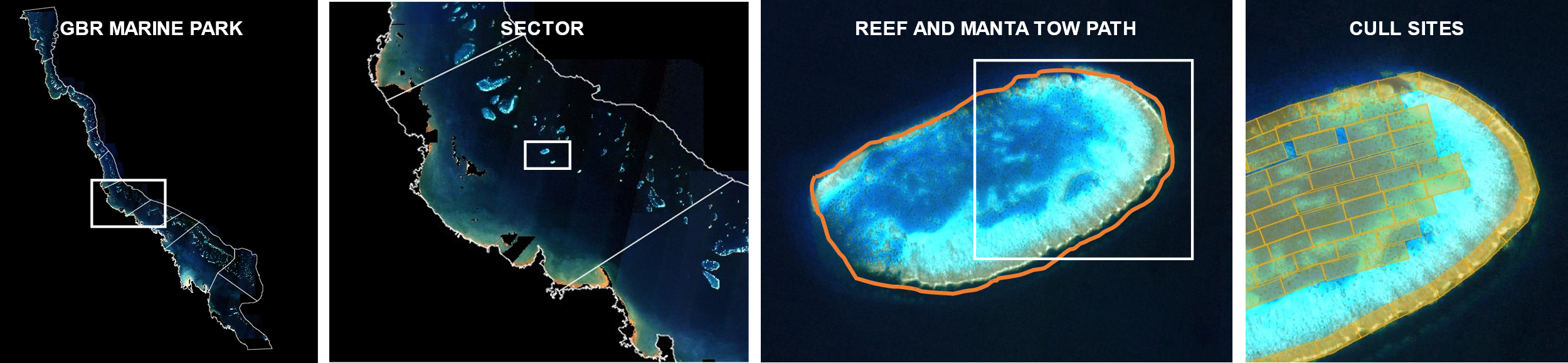


Fig S2. The spatial relationship between the Great Barrier Reef Marine Park (Marine Park), Sectors, Reefs, and Sites. **A)** the entire Marine Park, **B)** an individual Sector (Townsville), **C)** an individual reef (John Brewer Reef), with an indicative reef-wide manta-tow path as conducted by the AIMS LTMP **D)** culling sites. Satellite images obtained with permission from GBRMPA under a CC BY license, original copyright 2020. Sector outlines obtained with permission from AIMS under a CC BY license, original copyright 2023.


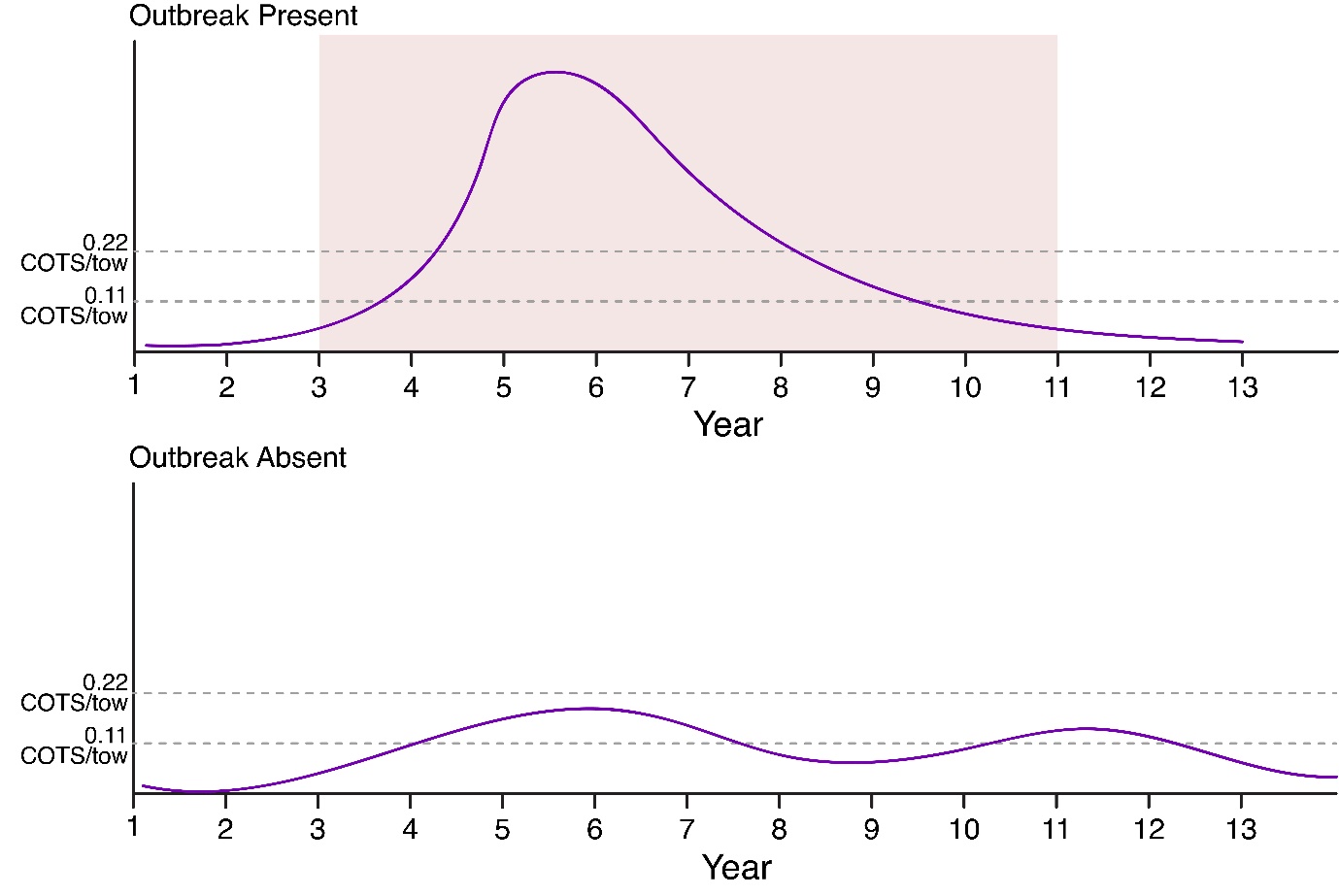


Fig S3. Graphical representation of sector-wide outbreak and non-outbreak populations. The purple line represents the COTS density at a given location. **Top** – An example of a population that would be categorised as an Outbreak. This is due to the population breaching the 0.22 COTS/tow density (an established outbreak). The years categorised as an ‘outbreak’ are denoted by the transparent, red rectangle. The year prior to the outbreak threshold being breached is included to capture the pre-outbreak coral cover and the outbreak is ended with two consecutive years below the threshold. **Bottom** – an example of a population that would not be categorised as an Outbreak. This is due to the density of COTS not crossing the 0.22 COTS/tow threshold (denoting a ‘severe’ outbreak). NB These sector-wide definitions of outbreak periods are distinct from the reef level outbreak which simply reflect the Outbreak Status of a reef at a singular point in time.


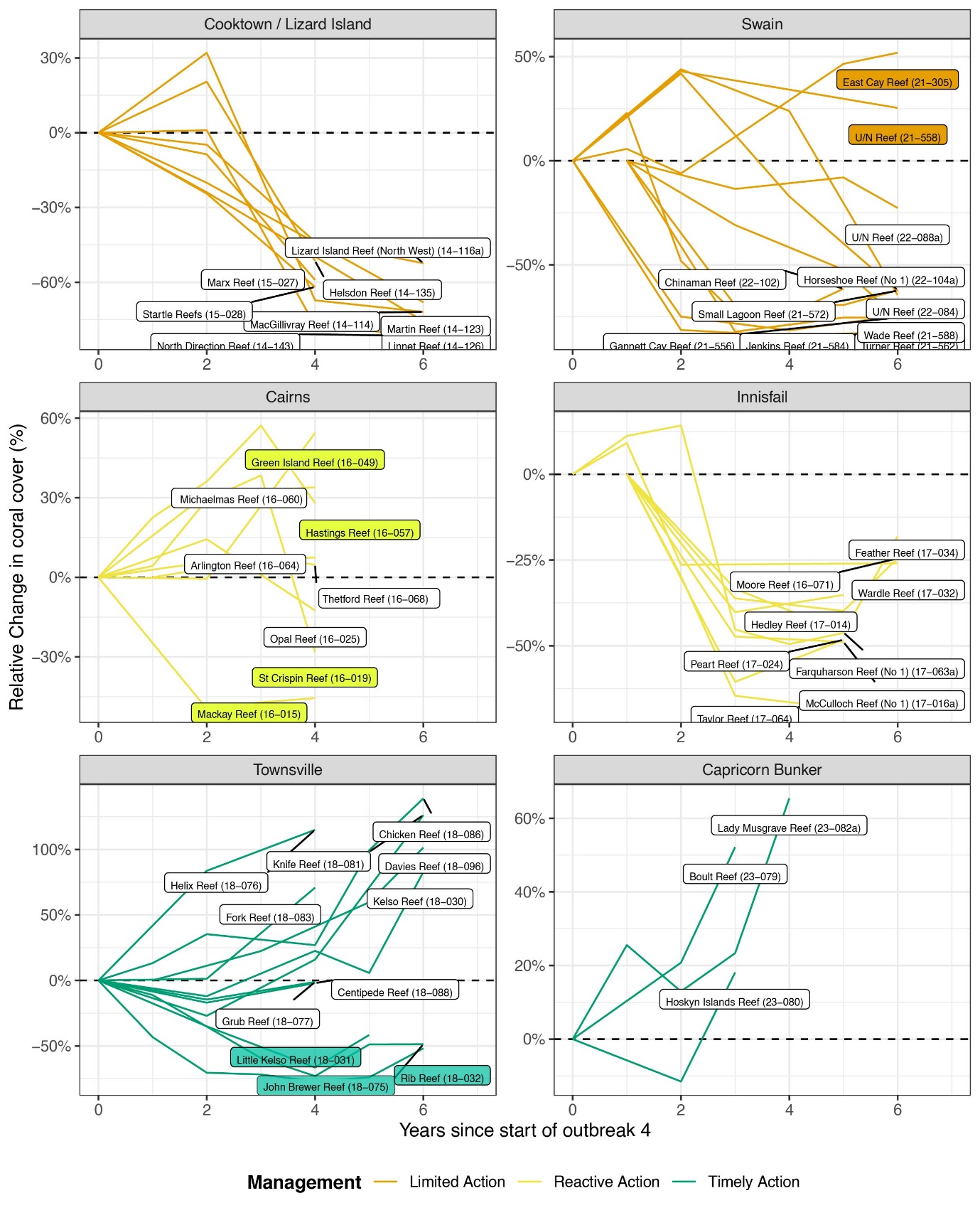


Fig S4. Relative change in coral cover (%) by Sector, coloured according to the type of management action implemented ‘Limited Action’ (orange), ‘Reactive’ (yellow), and ‘Timely’ (green) Each line represents the relative change in coral cover at a given reef, up to 6 years following the start of the sector-specific 4^th^ outbreak (see Table 1). Highlighted reefs are example outliers that are discussed in section 3.2. These individual trajectories underpin the modelled trajectory in Fig 4. NB Reefs surveyed less than 3 times were not included in this figure to increase the clarity of individual trajectories. Additionally each facet is displayed on a variable y-axis to increase visual interpretation. Reefs from “Proactive action” (Cape Upstart sector) are not included in either time series analyses (see Fig 4) as the time series is not long enough from the predicted onset of the outbreak in 2020.


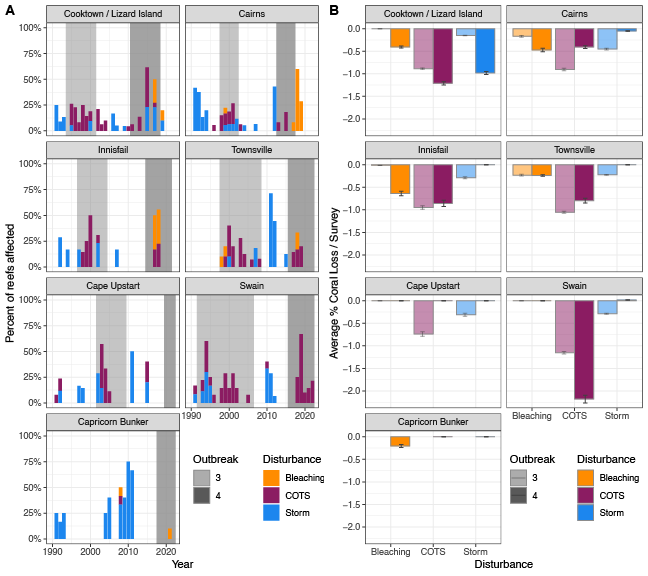


Fig S5. Disturbance regimes of the GBR by sector. (A) The percentage of reefs affected by the 3 major disturbances (COTS, Cyclones and Bleaching) from 1990 as observed by the AIMS LTMP broadscale manta tow surveys. Light and dark grey bars indicate the 3^rd^ and 4^th^ COTS outbreak waves respectively (B). The average coral loss per survey observed within the 3^rd^ and 4^th^ COTS outbreak waves for each of the three major disturbances.

## Supplementary Text

### Disturbance data

During AIMS LTMP manta tow observations, causes of mortality are attributed to significant coral losses (>5%) where possible. For Fig S5 (A) the percentage of reefs affected by each major disturbance (COTS, Bleaching, Storms) by sector was calculated each year using this attribution. For clarity, unknown disturbances were excluded as were coral disease due to the low prevalence in the dataset. For Fig S5 (B) the coral loss per survey was calculated as the mean +/- standard error of coral loss recorded from the year prior to the mortality event being observed for each disturbance type by sector. This metric includes all reefs that were surveyed and not just those affected by disturbance to provide a clear estimate of the prevalence and magnitude of coral losses from each disturbance.

### COTS control methods

COTS culling occurs in a highly organized and structured manner to ensure the most effective use of resources. Currently, multiple contractors (some with multiple vessels) perform culling following the Integrated Pest Management framework. This framework allows managers to make informed decisions about where to prioritise culling effort and when culling can cease at certain reefs.

Briefly put, each targeted reef is allocated to a specific contractor who exclusively perform the culling at that reef. First, the reef is surveyed using a manta tow during which a diver is pulled behind a small tender around the reef perimeter. Tows are broken into approximately 200m segments during which a diver records: 1) the estimated coral cover, 2) the number of COTS feeding scars, and 3) the number of COTS observed. This data is then paired with the GPS track recorded for each tow and is used to inform where culling should be performed.

Every actioned reef is broken into smaller management units called “sites” which are approximately 500m long and 200m wide. All culling is performed and recorded at the site level. Using a mix of snorkeling and SCUBA, a team of divers swim along the site in a line covering the full spread of reef habitats (slope, crest, and flat) and aim to completely survey the site within a fixed amount time (typically 40-60 minutes). Divers search for COTS and inject any located individuals with 10mL of ox bile solution or vinegar. This injection causes rapid disease formation and subsequent death in the following 24-48 hours. The number of COTS and their respective size cohort (<15cm, 15-25cm, 25-40cm, and >40cm) culled at each site is recorded after every dive. The catch per unit effort (CPUE: total number of COTS >15cm culled/total minutes spent culling) is calculated using the culling data for every site actioned. The CPUE is then used to determine if culling needs to be continued at that site (≥0.04, i.e. the site remains open) or if the site is then closed to culling (<0.04) as it now has an ecologically stable population of COTS.

All tow and culling data is uploaded and compiled in a central database after every voyage. This data is then made available to the contractors through an interactive dashboard that is referenced during subsequent voyages. Governing and management authorities also use this data to study trends and make informed decisions as to how to change or maintain the culling program.

### Categorising the Cape Upstart sector as ‘Proactive action’

The 4^th^ outbreak defined for the Cape Upstart sector is a forecast based upon historic outbreak cycles. The years that denote this outbreak are based on the southwardly progression of COTS and the observed 4-year delay between the 3^rd^ outbreak commencing in the Townsville sector (1998) and the Cape Upstart sector (2002). This expected outbreak was denoted only because this sector had demonstrated a history of previous outbreaks, thus allowing us to assume that there would be a current outbreak.

### ****Detailed definition of sector-wide management action****

1. ***Limited action:*** When limited resources are available and/or control activities were not initiated early enough COTS outbreaks may become too severe to control at both the reef and sector wide level. These reefs/sectors are typified by rapid coral loss beyond the resilience threshold with longer recovery times expected.
2. ***Reactive action:*** Control begins either after COTS outbreaks have exceeded severe outbreak levels or before this threshold is reached, but limited resources does not allow for the suppression of the outbreak. Substantially higher amounts of culling resources are required to reduce COTS numbers and prevent coral loss. Coral protection can thus only be achieved at a reduced number of sites and reefs. Coral protection may not be sustainable over the long term as continued COTS larval supply from surrounding reefs may eventually overwhelm control efforts. The goal of protection is to maintain coral cover above the resilience threshold (~10% coral cover, (63) at important source reefs to allow for increased rates of recovery following the outbreak (e.g., increased coral larvae export).
3. ***Timely action:*** Culling begins after reaching Potential outbreak densities >0.11 with sufficient resources to suppress the COTS populations. Suppression greatly reduces the regional spawning potential of COTS populations and a reduction in coral predation. This has positive consequences for both the culled reefs and those with downstream connectivity by reducing COTS larval supply and increasing coral larval supply.
4. ***Proactive action:*** Proactive action works via two mechanisms: pre-emptive culling targeted before COTS reach outbreak densities (0.11 COTS/Tow) and/or via effective suppression and containment in upstream sectors meaning that a sector wide outbreak does not eventuate. Importantly, proactive action takes place during the phase preceding the outbreak – the non-outbreak phase. The definition of proactive action via upstream suppression is reliant upon the sector in question having a history of sector-wide COTS outbreaks (working off the assumption that if culling did not occur, the sector would not be impacted by an outbreak). Successful proactive action at a reef/sector will have the greatest positive impact on coral growth and recovery. This approach will by definition result in lower total COTS culled and CPUE as efforts are targeted before outbreaks begin. It is important to note that proactive action in this context does not refer to COTS populations being eliminated from a sector. Instead, it simply means that a sector wide outbreak, which would have normally eventuated, has been prevented/delayed due to pre-emptive and/or upstream COTS control.

Table S1. Glossary of key words and acronyms used throughout the text with their definitions.

| **Term** | **Definition** |
| --- | --- |
| Threshold | A specific density of COTS that corresponds to altering important reef properties (e.g. 0.22 COTS/Tow) |
| Sector | Latitudinal bands used by AIMS to categorize reefs and track coral cover at broader scales. There are 11 sectors that span the entire length of the GBR which range in size from 7,500 – 55,000 km^2^. |
| Initiation Box | Reefs between Cairns and Lizard Island where primary outbreaks are believed to commence. |
| Primary Outbreak | The first outbreaks observed within the cycle. These arise independently from other outbreaks and occur on reefs within the initiation box (between Lizard Island and Cairns). |
| Secondary Outbreak | Successive outbreaks that result from increased larval supply from other outbreaks. Waves of secondary outbreaks generally move in a southward direction depending on prevailing currents. |
| GBRMPA | Great Barrier Reef Marine Park Authority. The governing body that manages all activities and operations within the marine park. |
| AIMS | Australian Institute of Marine Science. Government funded research institution focusing on tropical marine ecosystems in Australia. |
| LTMP | The AIMS Long-Term Monitoring Program. This is a large-scale assessment of coral cover on the entire GBR spanning four decades. |
| RRRC | The Reef and Rainforest Research Centre. One of the contractors that solicits operators to conduct the culling. |
| GBRF | The Great Barrier Reef Foundation. A partner that manages the funds used to hire contractors. |
| Severe Outbreak | When the average COTS/Tow for a reef exceeds 1 |
| Established Outbreak | When the average COTS/Tow for a reef range between 0.22 to 1 |
| Potential Outbreak | When the average COTS/Tow for a reef range between 0.11 to 0.22 |

Table S2. Model formulae and the description of the variables analysed.

| **model formula** | **Description** |
| --- | --- |
| COTS density ~ Sector*Outbreak + (1\|Reef) | COTS density as a product of the sector (a proxy for management objective) and outbreak (3^rd^ vs 4^th^) with the interaction thereof. The specific reef is included as a random effect. These results are presented in the main text. |
| Δ Coral Cover ~ Sector*Outbreak + (1\|Reef) | Relative change in coral cover as a product of the sector (a proxy for management objective) and outbreak (3^rd^ vs 4^th^) with the interaction thereof. The specific reef is included as a random effect. These results are presented in the main text. |
| Δ Coral Cover ~ s(Relative Year) | Relative change in coral cover as a smoothed product of the years since outbreak initiation within the sector. A model was fit to each sector individually and to each management objective. These results are presented in the main text. |
| Δ Coral Cover ~ Culling Effort + (1\|Sector) | Absolute change in coral cover as a product of the degree of culling effort applied. The sector from which the reef is from is included as a random effect. These results are presented in the main text. |

Table S4. Summary statistics for COTS densities and Coral Cover for each outbreak by sector. The 90% highest posterior density (HPD) intervals are given in parentheses (lower 90% : upper 90%)

|  |  | **COTS Densities** | | **Coral Cover** | |
| --- | --- | --- | --- | --- | --- |
|  | Sector | 3^rd^ Outbreak | 4^th^ Outbreak | 3^rd^ Outbreak | 4^th^ Outbreak |
|  | Cooktown-Lizard Island | 0.43 [0.23:0.69] | 0.90 [0.42:1.48] | -11% [-24:+4] | -41% [-58:-25] |
|  | Cairns | 0.20 [0.08:0.34] | 0.14 [0.04:0.32] | -11% [-29:+8] | +4% [-18:+28] |
|  | Innisfail | 0.69 [0.24:1.30] | 0.50 [0.18:1.02] | -33% [-53:-12] | -28% [-49:-7] |
|  | Townsville | 1.50 [0.52:2.68] | 0.21 [0.07:0.41] | -37% [-51:-20] | +44% [+23:+62] |
|  | Cape Upstart | 0.56 [0.09:1.45] | 0.11 [0.01:0.50] | -31% [-60:+1] | +14% [-33:+61] |
|  | Capricorn Bunker | NA | 0.25 [0.03:0.83] | NA | +46% [+4:+81] |
|  | Swain | 1.45 [0.39:3.18] | 1.72 [0.74:3.34] | -50% [-74:-27] | -46% [-68:-27] |
